# A multiscale computational model predicts distribution of anti-angiogenic isoform VEGF_165b_ in peripheral arterial disease in human and mouse

**DOI:** 10.1038/srep37030

**Published:** 2016-11-17

**Authors:** Liang-Hui Chu, Vijay Chaitanya Ganta, Min H. Choi, George Chen, Stacey D. Finley, Brian H. Annex, Aleksander S. Popel

**Affiliations:** 1 Department of Biomedical Engineering, School of Medicine, Johns Hopkins University, Baltimore, MD 21205, United States; 2 Cardiovascular Medicine, Department of Medicine, and the Robert M. Berne Cardiovascular Research Center University of Virginia School of Medicine, Charlottesville, VA 22901, United States; 3 Department of Biomedical Engineering, University of Southern California, Los Angeles, California 90089, United States.

## Abstract

Angiogenesis is the growth of new blood vessels from pre-existing microvessels. Peripheral arterial disease (PAD) is caused by atherosclerosis that results in ischemia mostly in the lower extremities. Clinical trials including VEGF-A administration for therapeutic angiogenesis have not been successful. The existence of anti-angiogenic isoform (VEGF_165b_) in PAD muscle tissues is a potential cause for the failure of therapeutic angiogenesis. Experimental measurements show that in PAD human muscle biopsies the VEGF_165b_ isoform is at least as abundant if not greater than the VEGF_165a_ isoform. We constructed three-compartment models describing VEGF isoforms and receptors, in human and mouse, to make predictions on the secretion rate of VEGF_165b_ and the distribution of various isoforms throughout the body based on the experimental data. The computational results are consistent with the data showing that in PAD calf muscles secrete mostly VEGF_165b_ over total VEGF. In the PAD calf compartment of human and mouse models, most VEGF_165a_ and VEGF_165b_ are bound to the extracellular matrix. VEGF receptors VEGFR1, VEGFR2 and Neuropilin-1 (NRP1) are mostly in ‘Free State’. This study provides a computational model of VEGF_165b_ in PAD supported by experimental measurements of VEGF_165b_ in human and mouse, which gives insight of VEGF_165b_ in therapeutic angiogenesis and VEGF distribution in human and mouse PAD model.

Angiogenesis is the process of new blood vessel formation from the pre-existing microvessels. Members of vascular endothelial growth factor (VEGF) superfamily critically but differentially regulate angiogenesis in normal physiological and pathophysiological conditions including exercise, ischemic cardiovascular diseases, and cancer^1^. The VEGF family includes five ligands VEGF-A, VEGF-B, VEGF-C, VEGF-D and PlGF (Placental growth factor), and five receptors VEGFR1, VEGFR2, VEGFR3, NRP1 (neuropilin-1) and NRP2 (neuropilin-2). Among the members of VEGF family, VEGF-A and VEGFR2 are considered to be potent pro-angiogenic molecules. However, recent identification of VEGF_xxxb_ isoforms has changed the classical paradigm of VEGF-A:VEGFR2 function in regulation of angiogenesis^2^.

Alternate splicing in the 8^th^ exon of VEGF-A results in the formation of sister families: pro-angiogenic VEGF_xxxa_ (VEGF_165a_, in human) isoform (xxx denotes number of amino acids) containing an amino acid sequence ‘CDKPRR’ and anti-angiogenic VEGF_165b_ isoform containing an amino acid sequence ‘PLTGKD’ in their C-terminus, respectively. The positively charged cysteine and arginine residues (CDKPRR) in pro-angiogenic VEGF-A isoform facilitate the binding of VEGF_165a_ to VEGFR2 and NRP1 to induce a conformational change and internal rotation of intracellular domain and maximal activation of VEGFR. However, replacement of cysteine and arginine residues with neutral lysine and aspartic acid in VEGF_xxxb_ isoform was predicted to result in partial VEGFR2 activation that cannot induce torsional rotation required for autophosphorylation and downstream signaling. Hence, the balance between VEGF_165a_ and VEGF_165b_ levels may play a crucial role in promoting angiogenesis especially in ischemic cardiovascular diseases such as peripheral arterial disease (PAD) or coronary artery disease (CAD).

PAD is caused by atherosclerosis, which results in ischemia most frequently in the lower extremities. Clinical trials including exogenous VEGF-A administration to activate VEGFR2 dependent therapeutic angiogenesis were not successful. While suboptimal delivery or dosage might be the contributing factors, induction of VEGF_165b_ in ischemic muscle could compete with pro-angiogenic VEGF_165a_ isoform for binding sites on VEGFR2 to decrease VEGFR2 activation. The mechanism of VEGF_165b_ binding to VEGFR2 suggests the potential reason for the failure of therapeutic angiogenesis in VEGF-A clinical trials. Currently, the balance between VEGF_165b_ and VEGF_165a_ isoforms that can modulate VEGFR2 activation and angiogenic signaling in the ischemic skeletal muscle of PAD patients is not fully understood.

We have previously reported experimental evidence that VEGF_165b_ levels are significantly higher in biopsies of PAD patients^3^. Kikuchi *et al*. measured the ratios of VEGF_165b_ versus VEGF_165a_ in serum (by western blot analysis) and peripheral blood mononuclear cells (PBMCs, by mRNA) in PAD patients, which are 4:1 and 8:1, respectively^4^. Currently there is no study measuring the ratio of VEGF_165b_ and VEGF_165a_ in the calf muscles to correlate with VEGFR2 activation in PAD patients. Besides, the understanding of the secretion rates of VEGF_165b_ in different compartments such as diseased calf muscles, blood and normal tissues (all tissues and organs except the diseased calf) is currently lacking. In this study, we expand the previously developed three-compartment models^5^,^6^ to predict the concentration of VEGF_165b_ and VEGF_165a_ in PAD patients. We also report the experimental measurements of the expression level of VEGF_165b_ and VEGF_164a_ in experimental PAD non-ischemic and ischemic muscle samples in mice with hindlimb ischemia to support our computational models.

## Results

In this study, we use both experimental and computational approaches to predict the secretion rate of VEGF_165b_ and receptor occupancy of VEGFR1 and VEGFR2 in PAD. In practice, it is not possible to measure the secretion rates of VEGF in different tissues *in vivo*. These important physiological parameters could be only estimated or calculated from the model. This is the first computational model to investigate and account for the experimental ratios of VEGF_165b_ and total VEGF_165_ in human PAD muscle biopsies. The predominance of VEGF_165b_ in human muscles provides the potential mechanism why the clinical trials of VEGF therapeutic angiogenesis have failed. Based on the measurement of ratio of VEGF_165b_ and total VEGF_165_ in muscle biopsies, we use the three-compartment model to predict the secretion rates of VEGF_165b_ in different tissues. This model enables prediction of receptor occupancy with VEGF_165b_ and other isoforms in human and mouse.

### Specificity and sensitivity of VEGF-A and VEGF_165b_ antibodies

**Figure 1:**
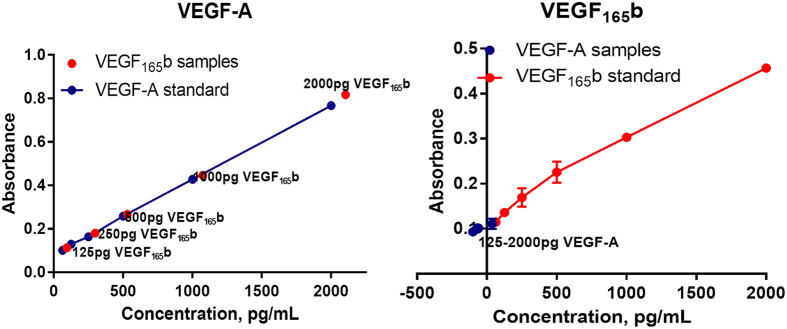
ELISA showing the specificity and sensitivity of VEGF_165a_ (VEGFA) and VEGF_165b_ antibodies.

VEGF-A ELISA detected both recombinant VEGF_165a_ and VEGF_165b_ with equal sensitivity and specificity. This result indicates that the actual measurement of pro-angiogenic VEGF-A by VEGF-A antibodies have been overestimated due to its cross reactivity with anti-angiogenic VEGF_165b_ isoforms. However, VEGF_165b_ antibody raised against the unique 6 amino acid sequence (SLTRKD) used in ELISA specifically detected only recombinant VEGF_165b_ but not recombinant VEGF_165a_ at any concentrations indicating that a ratio of VEGF_165b_:total VEGF_165_ measurements will be needed to determine the actual amount of pro-angiogenic VEGF-A isoforms in biological samples (Fig. 1).

### Three-compartmental models of PAD: motivation, assumptions and simulations

**Figure 2:**
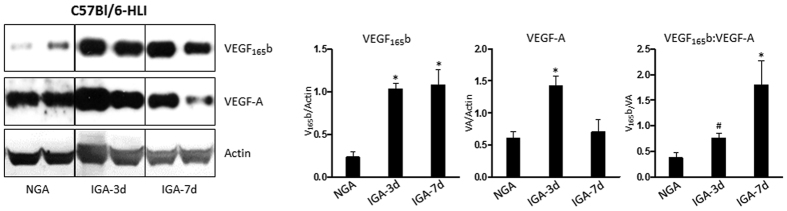
Western blot analysis of VEGF_165b_, VEGF-A and Actin in non-ischemic (NGA) and ischemic gastrocnemius muscle (IGA) at day 3 and day 7 post hindlimb ischemia (HLI). (n = 4. One-way ANOVA with Dunnetts post-test. p < 0.05 considered significant).

In our western analysis we observed a significant increase in total VEGF_165_ levels in ischemic muscle compared to non-ischemic at day 3 and day 7 post hindlimb ischemia (HLI). However, the levels of VEGF_165b_ were also significantly higher at day 3 and day 7 post HLI. The ratio VEGF_165b_:VEGF-A (from the densitometry values of western blot analysis) showed a ~4 fold induction in the fraction of VEGF_165b_ in total VEGF-A in ischemic muscle at day 7 post HLI compared to non-ischemic (i.e. VEGF_165b_ isoforms constitute ~80% of total VEGF-A) in experimental PAD muscle compared to control (Fig. 2).

We used the above experimental results in our model to predict the concentrations of VEGF_165b_ and VEGF_165a_ in the calf muscles. Kikuchi *et al*. measured the ratios of VEGF_165b_ versus VEGF_165a_ in serum (protein) and peripheral blood mononuclear cells (PBMCs, mRNA) in PAD patients, which were reported as 4:1 and 8:1, respectively^4^. Hoier *et al*. measured the interstitial total VEGF protein concentration (i.e. including both VEGF_165a_ and VEGF_165b_) in the thigh skeletal muscle of PAD patients as 69 ± 21 and 190 ± 78 pg/ml in rest and active exercise, respectively. We convert these numbers to molar concentrations as 69 pg/ml · 1000 ml/l · 1 mole/46000 g = 1.5 pM and 190 pg/ml = 4.1 pM, respectively, based on the molecular weight 46 kDa for VEGF homodimers^7^. We have constrained the model so that the predictions of steady-state concentration of VEGF_165b_ and total VEGF_165_ are within an experimentally observed range (e.g. 0–10 pM) in any of the three compartments to be consistent with the experimental data.

However, because of the sparseness of experimental data it is not possible to identify all the unknown parameters from the available data. Thus, we choose an initial set of secretion rates based on our previous analyses and perform the sensitivity analysis to determine a range of secretion rates constrained with available experimental data, both our own and others. Note that future experiments may provide additional data that could allow further constraining the solutions; presently, the analysis is heuristic aiming at stimulating further experiments.

### Prediction of secretion rates of VEGF_165b_ and secretion ratio of VEGF_165b_ over total VEGF in three compartments

The values of secretion rate of VEGF in normal and blood compartments are adopted from the tumor angiogenesis model^8^. The secretion rate in PAD calf compartment is set up the same as the secretion rate in the normal compartment. The initial set of secretion rates of total VEGF including VEGF_165a_, VEGF_165b_ and VEGF_121_ is assumed as 0.02, 0.031 and 0.02 molecules/cell/s, respectively, in all the three compartments (normal, blood and PAD calf). The secretion ratio of VEGF_121_ over total VEGF (VEGF_165a_ + VEGF_165b_ + VEGF_121_) is assumed to be 10%, i.e. 0.002, 0.0031 and 0.002 in normal, blood and disease compartments, respectively. We scan the ratio of secretion rate of VEGF_165b_ over total VEGF_165_ (i.e. the sum of VEGF_165a_ and VEGF_165b_) in the three compartments from 0 and 1 in 0.1 increments. When the ratio of VEGF_165b_ over total VEGF_165_ is 0.5 in all three compartments (i.e. secretion rates of VEGF_165a_ and VEGF_165b_ have equal values 0.009, 0.01395 and 0.009 in normal and disease compartments, respectively), the concentration of VEGF_165b_ in the normal compartment reaches 31 pM. When the ratio of VEGF_165b_ over total VEGF_165_ in all three compartments is 10%, the concentration of VEGF_165b_ in the normal compartment drops to 6.5 pM, which is within the experimentally observed range (<10 pM).

**Figure 3:**
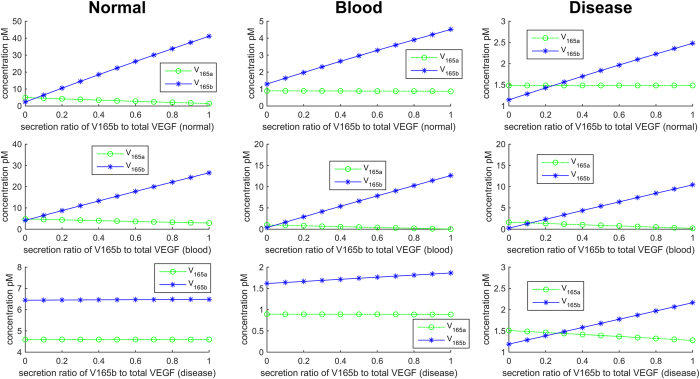
Concentrations of VEGF_165a_ and VEGF_165b_ plotted against the secretion ratio of VEGF_165b_ to total VEGF_165_ isoforms in normal (left column), blood (middle column) and disease compartments (right column). Each row represents the variation of secretion ratio of VEGF_165b_ to total VEGF_165_ in (**A**) normal, (**B**) blood, and (**C**) disease compartment.

We can also change the secretion ratio of VEGF_165b_ to total VEGF_165_ in each of the three compartments individually. We plot the predictions for steady-state concentrations of VEGF_165b_ and VEGF_165a_ in Fig. 3. We first change the secretion ratio of VEGF_165b_/total VEGF_165_ from 0 to 1 in 0.1 increments in the normal compartment while keeping the total secretion rate of VEGF_165_ (VEGF_165a_ + VEGF_165b_) constant at 0.018 molecules/cell/s in the normal and disease compartments and fixing the secretion ratios in blood and disease compartments at 0.1 (Fig. 3A). We follow the same strategy in Fig. 3B,C, except we vary the secretion ratio of VEGF_165b_/total VEGF_165_ in the blood compartment for B and the disease compartment for C, while the total secretion rates are fixed and the ratios are fixed at 0.1 in the other compartments. The model predicts that the concentration of VEGF_165b_ remains at approximately 6.5 pM, when the VEGF_165b_/total VEGF_165_ secretion ratio is 0.1 in normal and blood, across the full range of secretion ratios in the disease compartment (Fig. 3, left column). Additionally, the steady state concentration of VEGF_165b_ is consistently predicted to be approximately 30% higher than VEGF_165a_ in the disease compartment. This result is qualitatively consistent with our experimental data. This prediction gives us an important biological insight that the PAD disease calf muscles comprise mostly VEGF_165b_ over other total VEGF isoforms. This finding could be further extended for the development of VEGF_165b_ antibody treatment in PAD.

### Sensitivity analysis of VEGF_165a_, VEGF_165b_ and sVEGFR1

**Figure 4:**
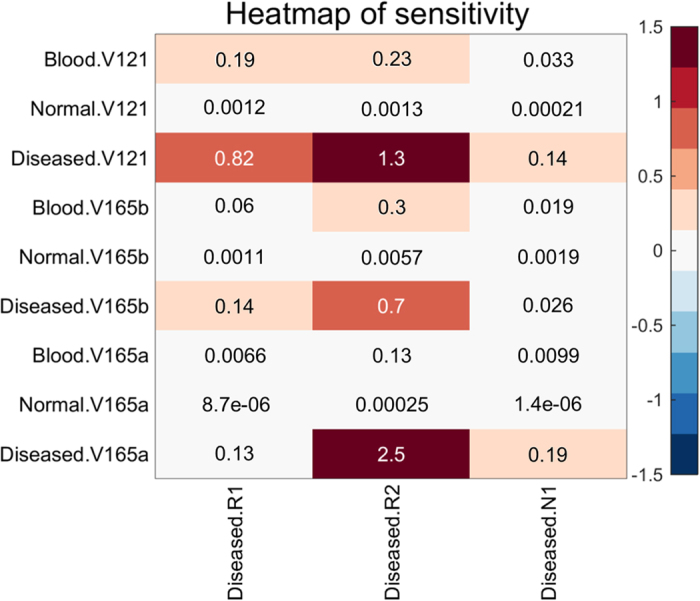
Local sensitivity analysis for VEGF_165a_, VEGF_165b_ and VEGF_121_. The results show that VEGF_165a_ in the disease compartment and VEGF_165b_ in the blood compartment are more sensitive to VEGFR2 than VEGFR1, whereas VEGF_121_ in the disease and blood compartment is sensitive to both VEGFR1 and VEGFR2. VEGF_165a_, VEGF_165b_, VEGF_121_ are not sensitive to NRP1. The sensitivity analysis demonstrates the importance of VEGF_165b_-VEGFR2 binding.

We use SimBiology to perform a local sensitivity analysis, which quantifies how changes in a given parameter value influence predicted concentrations of interest. We calculate the normalized sensitivity coefficient 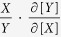 based on the algorithm^9^, where Y is the species VEGF_165a_, VEGF_165b_ and VEGF_121_, and X is the three VEGF receptors VEGFR1, VEGFR2 and NRP1 in the disease compartments; [X] and [Y] denote the molar concentrations. Figure 4 shows that VEGF_165a_ in the disease compartment and VEGF_165b_ in the blood compartment are more sensitive to VEGFR2 than VEGFR1, whereas VEGF_121_ in the disease and blood compartment is sensitive to both VEGFR1 and VEGFR2. VEGF_165a_, VEGF_165b_, VEGF_121_ are not sensitive to NRP1. The sensitivity analysis in Fig. 4 demonstrates the importance of VEGF_165b_-VEGFR2 binding.

### Tissue VEGF distribution and VEGFR occupancy in human three compartment model

**Figure 5:**
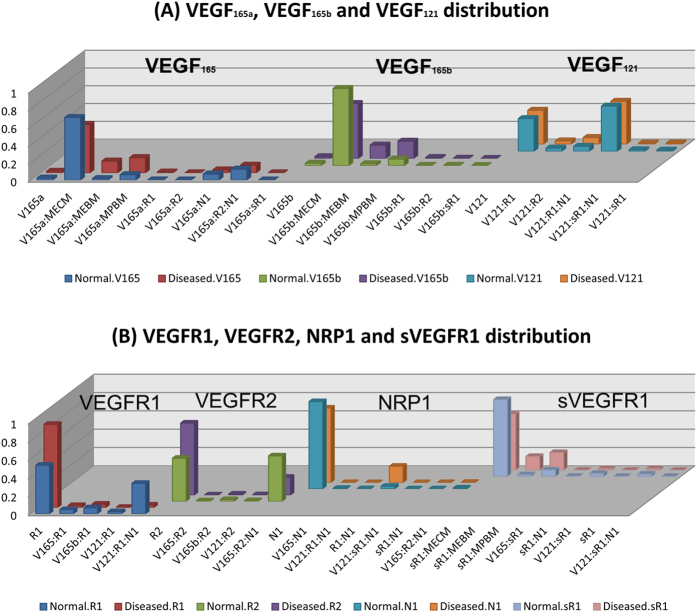
(**A**) VEGF distribution and (**B**) VEGFR occupancy in human. The bars in y-axis represent the percentage of each species for each VEGF ligand VEGF_165a_, VEGF_165b_ and VEGF_121_ in (**A**) and each receptor VEGFR1, VEGFR2, NRP1, and sVEGFR1 in (**B**).

We show the steady-state distribution of VEGF ligands and their receptors for each tissue in Fig. 5A,B, respectively. The y-axis represents the percentage of each species in x-axis in each VEGF ligand (VEGF_165a_, VEGF_165b_ and VEGF_121_) in Fig. 5A and each receptor (VEGFR1, VEGFR2, NRP1, and sVEGFR1) in Fig. 5B. In PAD calf, most VEGF_165a_ is bound to the ECM and parenchymal basement membrane (PBM) (55% and 17%, respectively); most VEGF_165b_ is also bound to ECM and PBM (62% and 20%, respectively). Here ECM represents the total extracellular matrix minus the endothelial and parenchymal basement membranes. Most VEGF_121_ isoform is bound to VEGFR1 and NRP1 as the VEGF_121_:VEGFR1:NRP1 complex in the disease (48%) and normal (51%) compartment. Regarding receptor occupancy, in the normal compartment, the three receptors VEGFR1, VEGFR2 and NRP1 are in the free states, except the VEGF_121_:VEGFR1:NRP1 (33%) and VEGF_165a_:VEGFR2:NRP1 (49%). In the PAD calf compartment, most receptors are in the free states, except the complexes VEGF_165a_:VEGFR2:NRP1 (20%) and VEGFR1:NRP1 (18%). Most sVEGFR1 are bound to ECM, endothelial basement membrane (EBM) and PBM (62%, 15% and 19%, respectively). Our model in PAD resembles the similar distribution of VEGF and VEGFR occupancy in tumor^10^.

### VEGF distribution and VEGFR occupancy in murine three compartment model

**Figure 6:**
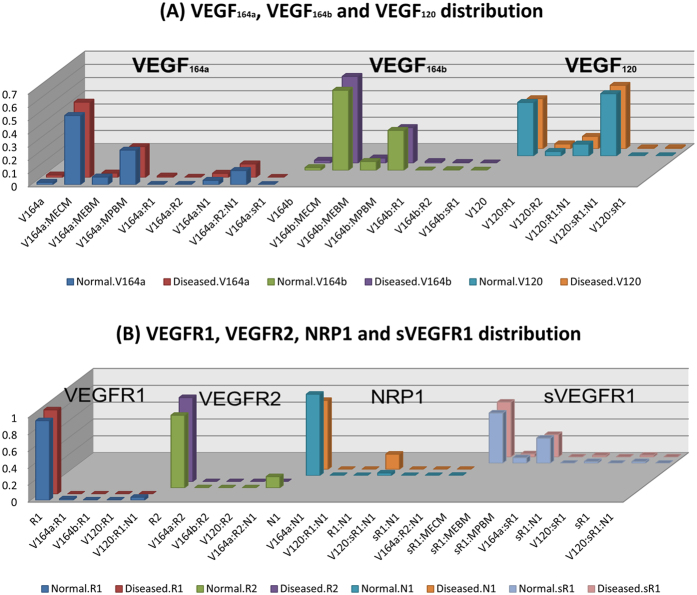
(**A**) VEGF distribution and (**B**) VEGFR occupancy in mouse. The bars represent the percentage of each species for each VEGF ligand in (**A**) and each receptor in (**B**), respectively, same as Fig. 5.

Constructing a computational model of mouse VEGF distribution is necessary to make use of the experimental measurements in hindlimb ischemia (HLI) models in mice where numerous studies have been conducted^3^,^11^,^12^. In the mouse, VEGF isoforms are shorter by one amino acid, e.g., VEGF_164a_, and VEGF_120_, but VEGF_165b_. Construction of the three-compartment model in mouse can help identify the relative concentration of VEGF_164a_ and VEGF_165b_ in the different compartments, and predict VEGF receptor occupancy. We replace the geometric parameters in the three-compartment model from human to mouse. These geometric parameters of mouse are described in the tumor xenograft model^6^ as well as in the mouse two-compartment model^13^. The results of VEGF distribution and VEGFR occupancy are shown in Fig. 6(A,B), respectively. Figures 5(A) and 6(A) show that VEGF_165b_ (human and mouse) mostly bind to the ECM and rarely exist as free ligands in both human and mouse. VEGF_164a_ and VEGF_165b_ bind to PBM with higher percentage in mouse than the corresponding isoforms in human. The VEGF receptor occupancies in Figs 5(B) and 6(B) show the similar trend between mouse and human. However, there is lower percentage of free VEGFR1 and VEGFR2 in human (Fig. 5B) than in mouse (Fig. 6B). This result is due to the higher percentage of VEGF_121_:VEGFR1:NRP1 and VEGF_165a_:VEGFR2:NRP1 in human than the corresponding complexes in mouse.

## Discussion

We previously developed the three-compartment models of VEGF in tumor^6^,^8^,^10^ and in PAD^14^. The anti-angiogenic form of VEGF_165b_ has not been included in any of the previous computational models; this is the first study to include VEGF_165b_ in our human PAD model. This is also the first study that compares human PAD and mouse hindlimb ischemia (HLI) models. By changing the geometric parameters in human PAD calf muscles and adding the kinetics equations of VEGF_165b_, we predict the VEGF_165b_ level in the PAD calf, blood and normal tissue compartments. We summarize the predictions in Table 1. Our computational model shows that the ratio of VEGF_165b_/VEGF_165a_ in PAD is higher than 1 (1.70, 2.10 and 1.41 in disease, blood and normal compartments, respectively). This prediction is consistent with the experimental measurements of VEGF_165b_ as the predominant isoform of VEGF in PAD^4^. The ratio of VEGF_165b_/total VEGF_165_ in the disease compartment of our model is slightly lower than the experiment in human biopsies; however, it should be noted that our predictions are for VEGF concentration in the interstitial fluid in the normal and diseased tissues, whereas the measurements represent the bulk tissue including intracellular components.

We do not include intrinsic heterogeneity that arises from the stochastic nature of biochemical reactions because we consider relatively large concentrations (pM). It may be more relevant to consider extrinsic heterogeneity (i.e., due to variations in protein concentrations)^15^. Others have included heterogeneity in the receptor distributions^16^ and found that variability in receptor expression can influence the response to anti-VEGF cancer treatment. The focus of the present work is to investigate and quantify how VEGF_165b_ molecular interactions influence overall VEGF distribution in human and mouse. This is the first model to incorporate the VEGF_165b_ isoform and we focus on the issues related to the expression of this isoform. Future work can incorporate variability in protein levels.

This study is also important for computational modeling of pharmacokinetics and pharmacodynamics (PK/PD) of administering VEGF_165b_-antibody as a potential pro-angiogenic therapeutic. Current VEGF antibodies including bevacizumab bind both total VEGF (VEGF_165a_, VEGF_165b_ and VEGF_121_). We propose to introduce an antibody to VEGF_165b_ as a PAD therapeutic to stimulate angiogenesis. This strategy will be explored in upcoming computational and experimental studies.

## Conclusions

We applied the three-compartment model of VEGF distribution to predict the concentration of VEGF isoforms, including VEGF_165b_, in the body in peripheral arterial disease. The experimental data show that the expression level of VEGF_165b_ is higher than VEGF_165a_ in the human biopsies and the computational model results are consistent with these measurements. The secretion ratio of VEGF_165b_ to total VEGF_165_ is estimated as 1 in the disease compartment, and 0.1 in the blood and normal compartments. Our predictions support the importance of the VEGF_165b_ in PAD, and provide a foundation for therapeutic inhibition of VEGF_165b_ in PAD in the future. This multiscale model can also provide a basis for simulating the pharmacokinetics of VEGF_165b_ antibody in PAD in human and mouse models.

## Methods

### ELISA to measure VEGF_165b_ antibody sensitivity

VEGF-A and VEGF_165b_ (R&D) Dual sandwich ELISAs were used to determine the sensitivity and specificity of VEGF-A and VEGF_165b_ antibodies in differentiating recombinant pro-angiogenic VEGF-A (VEGF_165a_) and anti-angiogenic VEGF-A (VEGF_165b_) isoforms. Recombinant VEGF_165a_ and VEGF_165b_ isoforms were serially diluted at 1000, 500, 250, 125 pg/ml concentrations and standard VEGF-A and VEGF_165b_ ELISA were performed according to manufacturer instructions on all the samples to determine the sensitivity and specificity of VEGF-A and VEGF_165b_ Mice.

### Murine Model of Hindlimb Ischemia

Hindlimb ischemia (HLI) induced by femoral artery ligation and resection was used as an experimental model of human PAD. All animal experiments were approved by the University of Virginia Animal Care and Use Committee and conformed to the Guide for the Care and Use of Laboratory Animals published by the US National Institutes of Health. HLI was performed on 8- to 12-week-old age and sex matched C57Bl/6 mice as described previously^11^,^12^. Briefly, mice were anesthetized by a combination of ketamine and Xylazine (ketamine 90 mg/kg and xylazine 10 mg/kg) and femoral artery was ligated and resected from just above the inguinal ligament to its bifurcation at the origin of saphenous and popliteal arteries. The inferior epigastric, lateral circumflex, and superficial epigastric artery branches were also ligated.

### Antibodies

VEGF_165b_ antibody was purchased from Millipore (Clone 56/1, Cat No: MABC595), VEGF-A antibody was purchased from Santa Cruz Biotech (Cat No: SC-7269) and β-Actin was purchased from Sigma (Cat No: A2103).

### Western blotting, Densitometry and Statistics

Mice were sacrificed with an overdose of anesthesia and non-ischemic and ischemic gastrocnemius muscle samples were collected at day 3 and day 7 post HLI. Tissue was homogenized in RIPA with protease inhibitor cocktail. Equal amounts of protein were resolved by Sodium Dodecyl Sulphate-Poly Acrylamide Gel Electrophoresis, transferred onto nitro cell cellulose membranes and western blotted against VEGF_165b_, VEGF-A and Actin by chemiluminescent method. Bands on the film were scanned and quantified by densitometric analysis of the band intensity by NIH image-J (1.6) software. Densitometric values were plotted on Graph PAD Prism 6 or 7 and analyzed for statistical significance. One-way ANOVA with Dunnetts post–test was used to check statistical significance. p < 0.05 considered statistically significant.

### Three-compartment models for human and mouse

**Figure 7:**
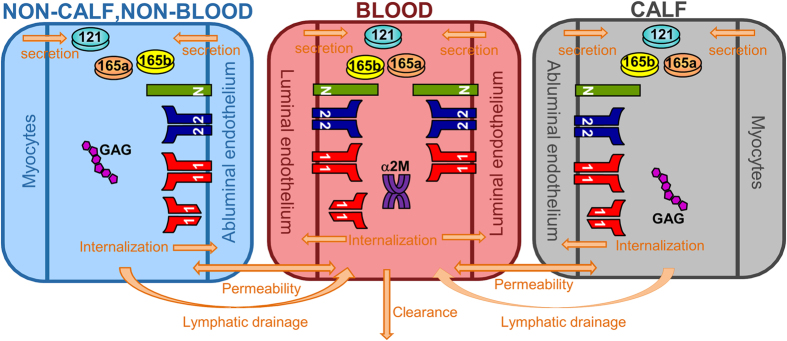
Three-compartment model of VEGF in peripheral arterial disease. 121: VEGF_121_. 165a: VEGF_165a_. 165b: VEGF_165b_. N: neuropilin-1. 1: soluble and membrane-bound VEGFR1. 2: VEGFR2. GAG: glycosaminoglycan. α2M: alpha-2 macroglobulin.

The model is comprised of three components: normal tissue (representing all tissues and organs except the diseased calf and blood), blood, and diseased calf of PAD (Fig. 7). For clarity, we use the notation VEGF_165a_ to denote the pro-angiogenic isoform; we refer to the combination of VEGF_165a_ and VEGF_165b_ as total VEGF_165_. VEGF_165a_, VEGF_165b_ and VEGF_121_ are secreted by myocytes and possibly stromal cells in the normal tissues and by calf muscles in PAD, respectively, and also by endothelial cells in all compartments. VEGF receptors (VEGFR1 and VEGFR2) and co-receptor neuropilin-1 (NRP1) are localized on the surfaces of endothelial and parenchymal cells. We include soluble VEGFR1 and glycosaminoglycan (GAG) chains in the interstitial space of normal and calf compartments, and alpha-2-macroglobulin (α2M) in the blood. These soluble factors bind VEGF and can be present in high concentrations; therefore, it is important to include them in the model. Geometric parameters are used to characterize the compartment and enable conversion of the concentrations from units used in the model (moles/cm^3^ tissue) to more standard units (molarity). The geometric parameters in the normal and blood human compartments are described in ref. 17 where applications to cancer are considered. The geometric parameters in the diseased calf compartment are described in ref. 14. The geometric parameters for mouse compartments are described in refs 6 and 13. We list the details of geometric parameters used in our model in Table 2.

### Binding of VEGF_165a_, VEGF_165b_ and VEGF_121_ to VEGFR1 and VEGFR2

**Figure 8:**
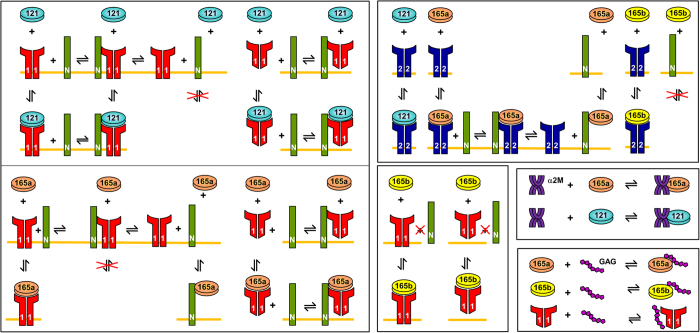
Molecular Interactions of VEGF_165a_, VEGF_165b_ and VEGF_121_.

The molecular interactions between VEGF isoforms and their receptors are illustrated in Fig. 8. VEGFR1 and VEGFR2 are the transmembrane receptors that bind to VEGF_165a_, VEGF_165b_ and VEGF_121_. VEGF_165b_ has the equivalent binding to VEGFR2 as VEGF_165a_^18^, and functions as a competitive inhibitor of the major downstream effects of VEGF_165a_. VEGF_165a_ and VEGF_165b_ are the two glycoproteins with heparin binding domain that can bind the extracellular matrix. VEGF_121_ is a freely diffusible protein lacking a heparin-binding domain. VEGF_165b_ could bind to VEGFR1 and VEGFR2 but cannot bind the co-receptor NRP1 because it lacks exon 8a^19^. The densities of cell receptors VEGFR1, VEGFR2 and NRP1 are listed in Table 3 based on available *in vivo* and *in vitro* experimental data. The kinetic parameters are listed in Table 4. The model is described in terms of 80 ordinary differential equations (ODE) and is presented in the Supplementary File.

We assume that the total secretion rate of VEGF isoforms to be 0.02 molecules/cells/s in the disease and normal compartment, and 0.031 in the blood compartment (see Results for the details). The model equations were implemented in MATLAB R2014b (MathWorks, Natick, MA) using the SimBiology toolbox and were solved with the Sundials solver.

### Transport parameters

Molecular species are transported between compartments via microvascular permeability (*k*_*p*_) and lymphatic drainage (*k*_*L*_) as shown in Fig. 7. All isoforms of unbound VEGF and soluble VEGFR1 (sVEGFR1) in the tissue compartments are subject to proteolytic degradation (*k*_*deg*_) and are removed from the blood via plasma clearance (*c*_*v*_). We list the transport parameters in Table 5.

### Sensitivity analysis

The sensitivity analysis is implemented using Matlab SimBiology toolbox. The time-dependent sensitivities of the species states are calculated with respect to species initial conditions and parameter values in the model. The sensitivity is calculated using 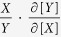, which determines how changes in parameter X influences the output Y by taking the partial derivatives.

## Additional Information

**How to cite this article**: Chu, L.-H. *et al*. A multiscale computational model predicts distribution of anti-angiogenic isoform VEGF_165b_ in peripheral arterial disease in human and mouse. *Sci. Rep.* **6**, 37030; doi: 10.1038/srep37030 (2016).

**Publisher’s note**: Springer Nature remains neutral with regard to jurisdictional claims in published maps and institutional affiliations.

## Supplementary Material

Supplementary Information

Supplementary Information

## Figures and Tables

**Table 1:** Prediction of VEGF_165b_, VEGF_165a_ and VEGF_121_.

Compartments	Diseased PAD calf (Total VEGF secretion rate: 0.02 molecules/cell/s)	Blood (Total VEGF secretion rate: 0.031 molecules/cell/s)	Normal (Total VEGF secretion rate: 0.02 molecules/cell/s)
Species			
VEGF_165a_	1.276 pM	0.886 pM	4.591 pM
VEGF_165b_	2.163 pM	1.863 pM	6.486 pM
VEGF_121_	0.701 pM	0.653 pM	2.416 pM

**Table 2:** Geometric parameters for the PAD calf compartment in human and mouse.

	Value	Units	Resources
Human PAD geometric parameters			
Compartment volume	738	cm^3^	Combined volume of lateral gastrocnemius, medial gastrocnemius, soleus muscles^20^
Fluid volume in ECM	31.87%	cm^3^/cm^3^ tissue	Calculated from ref. 14
Fluid volume in EBM	0.45%	cm^3^/cm^3^ tissue	Calculated from ref. 14
Fluid volume in PBM	0.58%	cm^3^/cm^3^ tissue	Calculated from ref. 14
Muscle fiber cell surface area	417	cm^2^/cm^3^ tissue	Calculated from ref. 14
Muscle fiber cross-sectional area	3,464	μm^2^	Calculated from refs 21, 22, 23
Mouse PAD geometric parameters			
Compartment volume	0.01	cm^3^	6
Fluid volume in ECM	28.92%	cm^3^/cm^3^ tissue	6
Fluid volume in EBM	0.097%	cm^3^/cm^3^ tissue	6
Fluid volume in PBM	0.68%	cm^3^/cm^3^ tissue	6
Muscle fiber cell surface area	713.68	cm^2^/cm^3^ tissue	Based on mouse myocytes^13^
Muscle fiber cross-sectional area	2500	μm^2^	Based on mouse myocytes^13^

**Table 3:** Number of cell surface receptors VEGFR1, VEGFR2 and NRP1.

Receptors	Value	Units	References
R1: Abluminal EC (normal)	3,750	receptors/EC	24,25
R2: Abluminal EC (normal)	300	receptors/EC	24,25
N1: Abluminal EC (normal)	20,000	receptors/EC	Extrapolated from receptor density on normal ECs, accounting for different cell surface areas
R1: Abluminal EC (Disease)	0	receptors/EC	24
R2: Abluminal EC (Disease)	0	receptors/EC	24
N1: Abluminal EC (Disease)	34,500	receptors/EC	24

Units of values: dimers/EC in VEGFR1 and VEGFR2 and dimer/EC in NRP1; EC: endothelial cell.

**Table 4:** Kinetic parameters.

	Value	unit	References
VEGF_165a_, VEGF_165b_ and VEGF_121_ binding to VEGFR1			
*k*_*on*_	3 × 10^7^	M^−1^ s^−1^	26,27
*k*_*off*_	10^−3^	s^−1^	26,27
*K*_*d*_	33	pM	26,27
VEGF_165a_, VEGF_165b_ and VEGF_121_ binding to VEGFR2			
*k*_*on*_	10^7^	M^−1^ s^−1^	26,27
*k*_*off*_	10^−3^	s^−1^	26,27
*K*_*d*_	100	pM	26,27
VEGF_165a_ and VEGF_121_ binding to NRP1			
*k*_*on*_	3.2 × 10^6^	M^−1^ s^−1^	26,27
*k*_*off*_	10^−3^	s^−1^	26,27
*K*_*d*_	312.5	pM	26,27
VEGF_165a_, VEGF_165b_ and VEGF_121_ binding to GAGs			
*k*_*on*_	4 × 10^5^	M^−1^ s^−1^	26,27
*k*_*off*_	10^−2^	s^−1^	26,27
*K*_*d*_	23.8	pM	26,27
VEGF_165a_, VEGF_165b_ and VEGF_121_ binding to α2M			
*k*_*on*_	25	M^−1^ s^−1^	Calculated
*k*_*off*_	10^−4^	s^−1^	Assumed
*K*_*d*_	4.0	μM	28
VEGF_165a_, VEGF_165b_ and VEGF_121_ binding to α2M_fast_			
*k*_*on*_	2.4 × 10^2^	M^−1^ s^−1^	Calculated
*k*_*off*_	10^−4^	s^−1^	Assumed
*K*_*d*_	0.42	μM	28
VEGF_165a_, VEGF_165b_ and VEGF_121_ binding to sVEGFR1			
*k*_*on*_	3 × 10^7^	M^−1^ s^−1^	Assume, based on VEGF binding to VEGFR1
*k*_*off*_	10^−3^	s^−1^	Assumed
*K*_*d*_	33	pM	Assumed
Coupling of NRP1 and VEGFR1			
*k*_*c*_	10^14^	(Mol/cm^2^)^−1^ s^−1^	26,27
*k*_*off*_	10^−2^	s^−1^	26,27
Coupling of NRP1 and VEGFR2			
*k*_*c V165R2*,*N1*_	3.1 × 10^13^	(Mol/cm^2^)^−1^ s^−1^	26,27
*k*_*off V165R2*,*N1*_	10^−3^	s^−1^	26,27
*k*_*c V165N1*,*R2*_	10^14^	(Mol/cm^2^)^−1^ s^−1^	26,27
*k*_*off V165N1*,*R2*_	10^−3^	s^−1^	26,27
sVEGFR1 binding to NRP1			
*k*_*on*_	5.6 × 10^6^	M^−1^ s^−1^	Calculated
*k*_*off*_	10^−2^	s^−1^	Assumed, based on VEGFR1 coupling to NRP1
*K*_*d*_	1.8	nM	29
sVEGFR1 binding to GAGs			
*k*_*on*_	4.2 × 10^5^	M^−1^ s^−1^	Assumed, based on VEGF binding to GAG
*k*_*off*_	10^−2^	s^−1^	Assumed
*K*_*d*_	24	pM	Assumed

**Table 5:** Transport parameters.

	Value	unit	References
Permeability between normal and blood compartments			
VEGF	4.0 × 10^−8^	cm/s	27
sVEGFR1	1.5 × 10^−8^	cm/s	30
VEGF:sVEGFR1 complex	1.5 × 10^−8^	cm/s	30
Permeability between disease and blood compartments			
VEGF	4.0 × 10^−7^	cm/s	Assumed, based on high permeability in PAD
sVEGFR1	3.0 × 10^−7^	cm/s	Assumed
VEGF:sVEGFR1 complex	1.5 × 10^−7^	cm/s	Assumed
Clearance			
VEGF	1.1 × 10^−3^	s^−1^	Calculated, based on half-life
sVEGFR1	5.0 × 10^−6^	s^−1^	30
VEGF:sVEGFR1	3.0 × 10^−4^	s^−1^	30
α2M	3.9 × 10^−5^	s^−1^	31
VEGF:α2M complex	3.9 × 10^−5^	s^−1^	Assumed, based on α2M
α2M_fast_	3.9 × 10^−3^	s^−1^	32
VEGF:α2M_fast_ complex	3.9 × 10^−3^	s^−1^	Assumed, based on α2M_fast_
Degradation			
sVEGFR1	1.9 × 10^−4^	s^−1^	Assumed, based on VEGF
VEGF:sVEGFR1 complex	1.9 × 10^−4^	s^−1^	Assumed, based on VEGF
Synthesis			
α2M	3.5 × 10^10^	Molecules/cm^3^ tissue/s	Calculated
α2M_fast_	1.9 × 10^10^	Molecules/cm^3^ tissue/s	Calculated
