# Supplementary material for: A multiscale computational model predicts distribution of anti-angiogenic isoform VEGF165b in peripheral arterial disease in human and mouse
Source: Sci Rep. 2016 Nov 17;6:37030. doi: 10.1038/srep37030 (PMC5113071; doi:10.1038/srep37030)
Supplement: Supplementary Information [file srep37030-s1.doc]

**A multiscale computational model predicts distribution of anti-angiogenic isoform VEGF_165b_ in peripheral arterial disease in human and mouse**

Liang-Hui Chu, Vijay Chaitanya Ganta, Min H. Choi, George Chen, Stacey D. Finley, Brian H. Annex, Aleksander S. Popel

**Supplementary material**

1. **Chemical reactions**

A system of 80 ordinary differential equations describes the temporal change of each molecular species' tissue and blood concentrations as a function of molecular binding interactions and transport processes. The relevant chemical reactions are presented here (molecular species and parameters are defined in the glossary):

**Receptor coupling**

**Soluble receptor**

1. **Equations for molecular species**

The complete list of ordinary differential equations is presented below:

**Normal Body and Calf Muscle PAD Tissue Equations**

I. Interstitial Matrix

$[M_{X}]$ denotes the concentration of unoccupied matrix binding sites in the ECM, EBM or PBM as specified by the subscript $X$. $[V_{165}]$ and $[V_{165b}]$ are the concentration of interstitial free $VEGF_{165}$ and $VEGF_{165b}$, respectively. $[\mathrm{sR}_{1}]$ is the concentration of interstitial free sVEGFR1. *i=N* for normal ECs and *i=D* for diseased calf muscle ECs.

(S. 1)

$\begin{matrix} \frac{d[M_{PBM}]_{i}}{dt}=-k_{on,V165,MPBM}^{i}[V_{165}]_{i}[M_{PBM}]_{i}+k_{off,V165MPBM}^{i}[V_{165}M_{PBM}]_{i} \\ -k_{on,V165b,MPBM}^{i}[V_{165b}]_{i}[M_{PBM}]_{i}+k_{off,V165bMPBM}^{i}[V_{165b}M_{PBM}]_{i} \\ -k_{on,sR1,MPBM}^{i}[sR_{1}]_{i}[M_{PBM}]_{i}+k_{off,sR1MPBM}^{i}[sR_{1}M_{PBM}]_{i} \end{matrix}$ (S. 2)

(S. 3)

$\frac{d[V_{165}M_{EBM}]_{i}}{dt}=k_{on,V165,MEBM}^{i}[V_{165}]_{i}[M_{EBM}]_{i}-k_{off,V165MEBM}^{i}[V_{165}M_{EBM}]_{i}$ (S. 4)

$\frac{d[V_{165}M_{PBM}]_{i}}{dt}=k_{on,V165,MPBM}^{i}[V_{165}]_{i}[M_{PBM}]_{i}-k_{off,V165MPBM}^{i}[V_{165}M_{PBM}]_{i}$ (S. 5)

$\frac{d[V_{165}M_{ECM}]_{i}}{dt}=k_{on,V165,MECM}^{i}[V_{165}]_{i}[M_{ECM}]_{i}-k_{off,V165MECM}^{i}[V_{165}M_{ECM}]_{i}$ (S. 6)

$\frac{d[V_{165b}M_{EBM}]_{i}}{dt}=k_{on,V165b,MEBM}^{i}[V_{165b}]_{i}[M_{EBM}]_{i}-k_{off,V165bMEBM}^{i}[V_{165b}M_{EBM}]_{i}$ (S. 7)

$\frac{d[V_{165b}M_{PBM}]_{i}}{dt}=k_{on,V165b,MPBM}^{i}[V_{165b}]_{i}[M_{PBM}]_{i}-k_{off,V165bMPBM}^{i}[V_{165b}M_{PBM}]_{i}$ (S. 8)

$\frac{d[V_{165b}M_{ECM}]_{i}}{dt}=k_{on,V165b,MECM}^{i}[V_{165b}]_{i}[M_{ECM}]_{i}-k_{off,V165bMECM}^{i}[V_{165b}M_{ECM}]_{i}$ (S. 9)

$\frac{d[sR_{1}M_{EBM}]_{i}}{dt}=k_{on,sR1,MEBM}^{i}[sR_{1}]_{i}[M_{EBM}]_{i}-k_{off,sR1MEBM}^{i}[sR_{1}M_{EBM}]_{i}$ (S. 10)

$\frac{d[sR_{1}M_{PBM}]_{i}}{dt}=k_{on,sR1,MPBM}^{i}[sR_{1}]_{i}[M_{PBM}]_{i}-k_{off,sR1MPBM}^{i}[sR_{1}M_{PBM}]_{i}$ (S. 11)

$\frac{d[sR_{1}M_{ECM}]_{i}}{dt}=k_{on,sR1,MECM}^{i}[sR_{1}]_{i}[M_{ECM}]_{i}-k_{off,sR1MECM}^{i}[sR_{1}M_{ECM}]_{i}$ (S.12)

II. Abluminal Endothelial Cell Surface

$[\mathrm{R}_{1}]$, $[\mathrm{R}_{2}]$ and $[\mathrm{N}_{1}]$ denote unoccupied VEGFR1, VEGFR2 and NRP1. $[\mathrm{V}\cdot\mathrm{R}_{1}]$, $[\mathrm{V}\cdot\mathrm{R}_{2}]$ and $[\mathrm{V}\cdot N_{1}]$ represent VEGF-bound VEGFR1, VEGFR2 and NRP1, respectively. $[\mathrm{R}\cdot\mathrm{V}\cdot N]$ and $[\mathrm{V}\cdot\mathrm{R}\cdot N]$ are NRP1-coupled VEGF-ligated VEGFRs. $k_{c}$ denoted the coupling rate between a VEGFR and the co-receptor NRP1. $k_{dissoc}$ denotes the direct decoupling of VEGFR and NRP1. $k_{\mathrm{int}}$ is the internalization of free or bound receptors, and $s_{R}$is the insertion rate of free receptors back in the cell membrane.

$\begin{matrix} \frac{d[R_{1}]_{i}}{dt}=s_{R1}^{i}-k_{on,V165,R1}^{i}[V_{165}]_{i}[R_{1}]_{i}+k_{off,V165R1}^{i}[V_{165}R_{1}]_{i} \\ -k_{on,V165b,R1}^{i}[V_{165b}]_{i}[R_{1}]_{i}+k_{off,V165bR1}^{i}[V_{165b}R_{1}]_{i} \\ -k_{on,V121,R1}^{i}[V_{121}]_{i}[R_{1}]_{i}+k_{off,V121R1}^{i}[V_{121}R_{1}]_{i} \\ -k_{c,R1,N1}^{i}[N_{1}]_{i}[R_{1}]_{i}+k_{dissoc,R1N1}^{i}[R_{1}N_{1}]_{i} \end{matrix}$ (S. 13)

$\begin{matrix} \frac{d[R_{2}]_{i}}{dt}=s_{R2}^{i}-k_{int,R2}^{i}[R_{2}]_{i}-k_{on,V121,R2}^{i}[V_{121}]_{i}[R_{2}]_{i}+k_{off,V121R2}^{N}[V_{121}R_{2}]_{i} \\ -k_{on,V165,R2}^{i}[V_{165}]_{i}[R_{2}]_{i}+k_{off,V165R2}^{i}[V_{165}R_{2}]_{i} \\ -k_{on,V165b,R2}^{i}[V_{165b}]_{i}[R_{2}]_{i}+k_{off,V165bR2}^{i}[V_{165b}R_{2}]_{i} \\ -k_{c,V165N1,R2}^{i}[V_{165}N_{1}]_{i}[R_{2}]_{i}+k_{off,V165N1,R2}^{i}[R_{2}V_{165}N_{1}]_{i} \end{matrix}$ (S. 14)

$\begin{matrix} \frac{d[N_{1}]_{i}}{dt}=s_{N1}^{i}-k_{int,N1}^{i}[N_{1}]_{i}-k_{c,V121R1,N1}^{i}[V_{121}R_{1}]_{i}[N_{1}]_{i}+k_{dissoc,V121R1N1}^{i}[V_{121}R_{1}N_{1}]_{i} \\ -k_{on,V165,N1}^{i}[V_{165}]_{i}[N_{1}]_{i}+k_{off,V165N1}^{N}[V_{165}N_{1}]_{i} \\ -k_{c,V165R2,N1}^{i}[V_{165}R_{2}]_{i}[N_{1}]_{i}+k_{off,V165R2N1}^{i}[V_{165}R_{2}N_{1}]_{i} \\ -k_{c,R1,N1}^{i}[N_{1}]_{i}[R_{1}]_{i}+k_{dissoc,R1N1}^{i}[R_{1}N_{1}]_{i} \end{matrix}$ (S. 15)

$\frac{d[V_{165}R_{1}]_{i}}{dt}=-k_{int,V165R1}^{i}[V_{165}R_{1}]_{i}+k_{on,V165,R1}^{i}[V_{165}]_{i}[R_{1}]_{i}-k_{off,V165R1}^{i}[V_{165}R_{1}]_{i}$ (S. 16)

$\begin{matrix} \frac{d[V_{165}R_{2}]_{i}}{dt}=-k_{int,V165R2}^{i}[V_{165}R_{2}]_{i}+k_{on,V165,R2}^{i}[V_{165}]_{i}[R_{2}]_{i}-k_{off,V165R2}^{i}[V_{165}R_{2}]_{i} \\ -k_{c,V165R2,N1}^{i}[V_{165}R_{2}]_{i}[N_{1}]_{i}+k_{off,V165R2N1}^{i}[R_{2}V_{165}N_{1}]_{i} \end{matrix}$ (S. 17)

$\frac{d[V_{165b}R_{1}]_{i}}{dt}=-k_{int,V165bR1}^{i}[V_{165b}R_{1}]_{i}+k_{on,V165b,R1}^{i}[V_{165b}]_{i}[R_{1}]_{i}-k_{off,V165bR1}^{i}[V_{165b}R_{1}]_{i}$ (S. 18)

$\frac{d[V_{165b}R_{2}]_{i}}{dt}=-k_{int,V165bR2}^{i}[V_{165b}R_{2}]_{i}+k_{on,V165b,R2}^{i}[V_{165b}]_{i}[R_{2}]_{i}-k_{off,V165bR2}^{i}[V_{165b}R_{2}]_{i}$ (S. 19)

$\begin{matrix} \frac{d[V_{165}N_{1}]_{i}}{dt}=-k_{int,V165N1}^{i}[V_{165}N_{1}]_{i}+k_{on,V165,N1}^{i}[V_{165}]_{i}[N_{1}]_{i}-k_{off,V165N1}^{i}[V_{165}N_{1}]_{i} \\ -k_{c,V165N1,R2}^{i}[V_{165}N_{1}]_{i}[R_{2}]_{i}+k_{off,V165N1R2}^{i}[R_{2}V_{165}N_{1}]_{i} \end{matrix}$ (S. 20)

$\begin{matrix} \frac{d[R_{2}V_{165}N_{1}]_{i}}{dt}=-k_{int,V165R2N1}^{i}[R_{2}V_{165}N_{1}]_{i} \\ +k_{c,V165R2,N1}^{i}[V_{165}R_{2}]_{i}[N_{1}]_{i}-k_{off,V165R2N1}^{i}[R_{2}V_{165}N_{1}]_{i} \\ +k_{c,V165N1,R2}^{i}[V_{165}N_{1}]_{i}[R_{2}]_{i}-k_{off,V165N1R2}^{i}[R_{2}V_{165}N_{1}]_{i} \end{matrix}$ (S. 21)

$\begin{matrix} \frac{d[V_{121}R_{1}]_{i}}{dt}=-k_{int,V121R1}^{i}[V_{121}R_{1}]_{i} \\ +k_{on,V121,R1}^{i}[V_{121}]_{i}[R_{1}]_{i}-k_{off,V121R1}^{i}[V_{121}R_{1}]_{i} \\ -k_{c,R1,N1}^{i}[V_{121}R_{1}]_{i}[N_{1}]_{i}+k_{dissoc,R1N1}^{i}[V_{121}R_{1}N_{1}]_{i} \end{matrix}$ (S. 22)

$\frac{d[V_{121}R_{2}]_{i}}{dt}=-k_{\text{int,V121R2}}^{i}[V_{121}R_{2}]_{i}+k_{on,V121,R2}^{i}[V_{121}]_{i}[R_{2}]_{i}-k_{off,V121R2}^{i}[V_{121}R_{2}]_{i }$ (S. 23)

$\begin{matrix} \frac{d[V_{121}R_{1}N_{1}]_{i}}{dt}=-k_{int,V121R1N1}^{i}[V_{121}R_{1}N_{1}]_{i} \\ +k_{c,V121R1,N1}^{i}[V_{121}R_{1}]_{i}[N_{1}]_{i}-k_{dissoc,V121R1N1}^{i}[V_{121}R_{1}N_{1}]_{i} \\ +k_{on,V121,R1N1}^{i}[V_{121}]_{i}[R_{1}N_{1}]_{i}-k_{off,V121R1N1}^{i}[V_{121}R_{1}N_{1}]_{i} \end{matrix}$ (S. 24)

$\begin{matrix} \frac{d[R_{1}N_{1}]_{i}}{dt}=-k_{int,R1N1}^{i}[R_{1}N_{1}]_{i} \\ +k_{c,R1,N1}^{i}[R_{1}]_{i}[N_{1}]_{i}-k_{dissoc,R1N1}^{i}[R_{1}N_{1}]_{i} \\ -k_{on,V121,R1}^{i}[V_{121}]_{i}[R_{1}N_{1}]_{i}+k_{off,V121R1}^{i}[V_{121}R_{1}N_{1}]_{i} \end{matrix}$ (S. 25)

$\begin{matrix} \frac{d[V_{121}sR_{1}N_{1}]_{i}}{dt}=-k_{int,V121sR1N1}^{i}[V_{121}sR_{1}N_{1}]_{i} \\ +k_{on,sR1,N1}^{i}[V_{121}R_{1}]_{i}[N_{1}]_{i}-k_{off,sR1N1}^{i}[V_{121}sR_{1}N_{1}]_{i} \\ +k_{on,V121sR1N1}^{i}[V_{121}]_{i}[R_{1}N_{1}]_{i}-k_{off,V121sR1N1}^{i}[V_{121}sR_{1}N_{1}]_{i} \end{matrix}$ (S. 26)

$\begin{matrix} \frac{d[sR_{1}N_{1}]_{i}}{dt}=-k_{int,sR1N1}^{i}[sR_{1}N_{1}]_{i}+k_{on,sR1,N1}^{i}[sR_{1}]_{i}[N_{1}]_{i}-k_{off,sR1N1}^{i}[sR_{1}N_{1}]_{i} \\ -k_{on,V121,sR1N1}^{i}[V_{121}]_{i}[sR_{1}N_{1}]_{i}+k_{off,V121,sR1N1}^{i}[V_{121}sR_{1}N_{1}]_{i} \end{matrix}$ (S. 27)

III. Interstitial Fluid

The interstitial concentrations of the complexes formed between the VEGF isoforms and sVEGFR1 are denoted $[V\cdot sR_{1}]_{N}$. Free VEGF isoforms are secreted at constant rates of $\mathrm{q}_{\mathrm{V}}$ from myocytes, while the free sVEGFR1 is secreted by endothelial cells at a constant rate of $\mathrm{q}_{sR1}$. All interstitial soluble species are subject to lymphatic drainage into the blood at a rate of $k_{L}$. The bidirectional vascular permeability flow at rates of $k_{p,V}^{ij}$ represents the microvascular permeability of VEGF from compartment *i* to compartment *j* (N=normal tissue, B=blood, D=PAD calf). $S_{i\mathrm{B}}$ denotes the total abluminal endothelial surface area exposed to the interstitial space of tissue *i*. *U_i_* is the volume of compartment *i* (*N*=normal tissue, *B*=blood, *D*=PAD calf). The geometric conversion factors ($S_{iB}$,$U_{i}$ and $K_{AV}$) confine the macromolecular exchange volumes to the available interstitial fluid volume in the tissue compartments and the plasma volume in the blood compartment.

$\begin{matrix} \frac{d[V_{165}]_{i}}{dt}=q_{V165}^{i}-k_{\deg,V}[V_{165}]_{i} \\ -k_{on,V165,MEBM}^{i}[V_{165}]_{i}[M_{EBM}]_{i}+k_{off,V165,MEBM}^{i}[V_{165}M_{EBM}]_{i} \\ -k_{on,V165,MPBM}^{i}[V_{165}]_{i}[M_{PBM}]_{i}+k_{off,V165,MPBM}^{i}[V_{165}M_{PBM}]_{i} \\ -k_{on,V165,MECM}^{i}[V_{165}]_{i}[M_{ECM}]_{i}+k_{off,V165,MECM}^{i}[V_{165}M_{ECM}]_{i} \\ -k_{on,V165,R1}^{i}[V_{165}]_{i}[R_{1}]_{i}+k_{off,V165R1}^{i}[V_{165}R_{1}]_{i} \\ -k_{on,V165,R2}^{i}[V_{165}]_{i}[R_{2}]_{i}+k_{off,V165R2}^{i}[V_{165}R_{2}]_{i} \\ -k_{on,V165,N1}^{i}[V_{165}]_{i}[N_{1}]_{i}+k_{off,V165N1}^{i}[V_{165}N_{1}]_{i} \\ -\left(\frac{k_{L}+k_{p,V}^{iB}S_{iB}}{U_{i}}\right)\frac{[V_{165}]_{i}}{K_{AV,\mathrm{i}}}+k_{p,V}^{Bi}\frac{S_{iB}}{U_{i}}\frac{U_{B}}{U_{P}}[V_{165}]_{B} \end{matrix}$ (S. 28)

$\begin{matrix} \frac{d[V_{165b}]_{i}}{dt}=q_{V165b}^{i}-k_{\deg,V}[V_{165b}]_{i} \\ -k_{on,V165b,MEBM}^{i}[V_{165b}]_{i}[M_{EBM}]_{i}+k_{off,V165b,MEBM}^{i}[V_{165b}M_{EBM}]_{i} \\ -k_{on,V165b,MPBM}^{i}[V_{165b}]_{i}[M_{PBM}]_{i}+k_{off,V165b,MPBM}^{i}[V_{165b}M_{PBM}]_{i} \\ -k_{on,V165b,MECM}^{i}[V_{165b}]_{i}[M_{ECM}]_{i}+k_{off,V165b,MECM}^{i}[V_{165b}M_{ECM}]_{i} \\ -k_{on,V165b,R1}^{i}[V_{165b}]_{i}[R_{1}]_{i}+k_{off,V165bR1}^{i}[V_{165b}R_{1}]_{i} \\ -k_{on,V165b,R2}^{i}[V_{165b}]_{N}[R_{2}]_{N}+k_{off,V165bR2}^{i}[V_{165b}R_{2}]_{i} \\ -\left(\frac{k_{L}+k_{p,V}^{iB}S_{iB}}{U_{i}}\right)\frac{[V_{165b}]_{i}}{K_{AV,\mathrm{i}}}+k_{p,V}^{Bi}\frac{S_{iB}}{U_{i}}\frac{U_{B}}{U_{P}}[V_{165b}]_{B} \end{matrix}$ (S. 29)

$\begin{matrix} \frac{d[V_{121}]_{i}}{dt}=q_{V121}^{i}-k_{\deg,V}[V_{121}]_{i}-k_{on,V121,R1}^{i}[V_{121}]_{i}[R_{1}]_{i}+k_{off,V121R1}^{i}[V_{121}R_{1}]_{i} \\ -k_{on,V121,R1N1}^{i}[V_{121}]_{i}[R_{1}N_{1}]_{i}+k_{off,V121R1N1}^{i}[V_{121}R_{1}N_{1}]_{i} \\ -k_{on,V121,R2}^{i}[V_{12}]_{i}[R_{2}]_{i}+k_{off,V121R2}^{i}[V_{121}R_{2}]_{i} \\ -\left(\frac{k_{L}+k_{p,V}^{iB}S_{iB}}{U_{i}}\right)\frac{[V_{121}]_{i}}{K_{AV,\mathrm{i}}}+k_{p,V}^{Bi}\frac{S_{iB}}{U_{i}}\frac{U_{B}}{U_{P}}[V_{121}]_{B} \end{matrix}$ (S. 30)

$\begin{matrix} \frac{d[sR_{1}]_{i}}{dt}=q_{sR1}^{i}-k_{\deg,sR1}^{i}[sR_{1}]_{i} \\ -k_{on,sR1,MEBM}^{i}[sR_{1}]_{i}[M_{EBM}]_{N}+k_{off,sR1,MEBM}^{i}[sR_{1}M_{EBM}]_{i} \\ -k_{on,sR1,MPBM}^{i}[sR_{1}]_{i}[M_{PBM}]_{N}+k_{off,sR1,MPBM}^{i}[sR_{1}M_{PBM}]_{i} \\ -k_{on,sR1,MECM}^{i}[sR_{1}]_{i}[M_{ECM}]_{N}+k_{off,sR1,MECM}^{i}[sR_{1}M_{ECM}]_{i} \\ -k_{on,V165,sR1}^{i}[V_{165}]_{i}[sR_{1}]_{i}+k_{off,V165sR1}^{i}[V_{165}sR_{1}]_{i} \\ -k_{on,V165b,sR1}^{i}[V_{165b}]_{i}[sR_{1}]_{i}+k_{off,V165bsR1}^{i}[V_{165b}sR_{1}]_{i} \\ -k_{on,V121,sR1}^{i}[V_{121}]_{i}[sR_{1}]_{i}+k_{off,V121sR1}^{i}[V_{121}sR_{1}]_{i} \\ -k_{on,sR1,N1}^{i}[sR_{1}]_{i}[N_{1}]_{i}+k_{off,sR1N1}^{i}[sR_{1}N_{1}]_{i} \\ -\left(\frac{k_{L}+k_{p,sR1}^{iB}S_{iB}}{U_{i}}\right)\frac{[sR_{1}]_{i}}{K_{AV,\mathrm{i}}}+k_{p,sR1}^{Bi}\frac{S_{iB}}{U_{i}}\frac{U_{B}}{U_{P}}[sR_{1}]_{B} \end{matrix}$ (S. 31)

$\begin{matrix} \frac{d[V_{165}sR_{1}]_{i}}{dt}=-k_{\deg,VsR1}^{i}[V_{165}sR_{1}]_{i}+k_{on,V165,sR1}^{i}[V_{165}]_{i}[sR_{1}]_{i}-k_{off,V165sR1}^{i}[V_{165}sR_{1}]_{i} \\ -\left(\frac{k_{L}+k_{p,VsR1}^{iB}S_{iB}}{U_{i}}\right)\frac{[V_{165}sR_{1}]_{i}}{K_{AV,\mathrm{i}}}+k_{p,VsR1}^{Bi}\frac{S_{iB}}{U_{i}}\frac{U_{B}}{U_{P}}[V_{165}sR_{1}]_{B} \end{matrix}$ (S. 32)

$\begin{matrix} \frac{d[V_{165b}sR_{1}]_{i}}{dt}=-k_{\deg,VsR1}^{i}[V_{165b}sR_{1}]_{i}+k_{on,V165b,sR1}^{i}[V_{165b}]_{i}[sR_{1}]_{i}-k_{off,V165bsR1}^{i}[V_{165b}sR_{1}]_{i} \\ -\left(\frac{k_{L}+k_{p,VsR1}^{iB}S_{iB}}{U_{i}}\right)\frac{[V_{165b}sR_{1}]_{i}}{K_{AV,i}}+k_{p,VsR1}^{Bi}\frac{S_{iB}}{U_{i}}\frac{U_{B}}{U_{P}}[V_{165b}sR_{1}]_{B} \end{matrix}$ (S. 33)

$\begin{matrix} \frac{d[V_{121}sR_{1}]_{i}}{dt}=-k_{\deg,VsR1}^{i}[V_{121}sR_{1}]_{i} \\ +k_{on,V121,sR1}^{i}[V_{121}]_{i}[sR_{1}]_{i}-k_{off,V121sR1}^{i}[V_{121}sR_{1}]_{i} \\ -k_{on,sR1,N1}^{i}[V_{121}sR_{1}]_{i}[N_{1}]_{i}+k_{off,R1sN1}^{i}[V_{121}sR_{1}N_{1}]_{i} \\ -\left(\frac{k_{L}+k_{p,VsR1}^{iB}S_{iB}}{U_{i}}\right)\frac{[V_{121}sR_{1}]_{i}}{K_{AV,\mathrm{i}}}+k_{p,VsR1}^{Bi}\frac{S_{iB}}{U_{i}}\frac{U_{B}}{U_{P}}[V_{121}sR_{1}]_{B} \end{matrix}$ (S. 34)

**Blood compartment equations**

We denote the luminal receptors and ligand-receptor complexes on endothelial cells (ECs) by the subscript *i* (*i=N* for normal ECs; *i=D* for diseased calf muscle ECs).

I. Luminal side of normal/ calf endothelium

$\begin{matrix} \frac{d[R_{1}]_{B,i}}{dt}=s_{R1}^{B}-k_{int,R1}^{B}[R_{1}]_{B,i}-k_{on,V165,R1}^{B} [V_{165}]_{B}[R_{1}]_{B,i}+k_{off,V165R1}^{B}[V_{165}R_{1}]_{B,i} \\ -k_{on,V165b,R1}^{B} [V_{165b}]_{B}[R_{1}]_{B,i}+k_{off,V165bR1}^{B}[V_{165b}R_{1}]_{B,i} \\ -k_{on,V121,R1}^{B}[V_{121}]_{B}[R_{1}]_{B,i}+k_{off,V121R1}^{B}[V_{121}R_{1}]_{B,i} \\ -k_{c,R1,N1}^{B}[N_{1}]_{B,i}[R_{1}]_{B,i}+k_{dissoc,R1N1}^{B}[R_{N}N_{1}]_{B,i} \\ -k_{on,V165,R1}^{B} [\alpha2M\cdot V_{165}]_{B}[R_{1}]_{B,i}+k_{off,V165R1}^{B}[\alpha2M\cdot V_{165}R_{1}]_{B,i} \\ -k_{on,V165b,R1}^{B} [\alpha2M\cdot V_{165b}]_{B}[R_{1}]_{B,i}+k_{off,V165bR1}^{B}[\alpha2M\cdot V_{165b}R_{1}]_{B,i} \\ -k_{on,V121,R1}^{B}[\alpha2M\cdot V_{121}]_{B}[R_{1}]_{B,i}+k_{off,V121R1}^{B}[\alpha2M\cdot V_{121}R_{1}]_{B,i} \\ -k_{c,R1,N1}^{B}[\alpha2M\cdot N_{1}]_{B,i}[R_{1}]_{B,i}+k_{dissoc,R1N1}^{B}[\alpha2M\cdot R_{1}N_{1}]_{B,i} \\ -k_{on,V165,R1}^{B} [\alpha2M_{fast}\cdot V_{165}]_{B}[R_{1}]_{B,i}+k_{off,V165R1}^{B}[\alpha2M_{fast}\cdot V_{165}R_{1}]_{B,i} \\ -k_{on,V165b,R1}^{B} [\alpha2M_{fast}\cdot V_{165b}]_{B}[R_{1}]_{B,i}+k_{off,V165bR1}^{B}[\alpha2M_{fast}\cdot V_{165b}R_{1}]_{B,i} \\ -k_{on,V121,R1}^{B}[\alpha2M_{fast}\cdot V_{121}]_{B}[R_{1}]_{B,i}+k_{off,V121R1}^{B}[\alpha2M_{fast}\cdot V_{121}R_{1}]_{B,i} \\ -k_{c,R1,N1}^{B}[\alpha2M_{fast}\cdot N_{1}]_{B,i}[R_{1}]_{B,i}+k_{dissoc,R1N1}^{B}[\alpha2M_{fast}\cdot R_{1}N_{1}]_{B,i} \end{matrix}$ (S. 35)

$\begin{matrix} \frac{d[R_{2}]_{B,i}}{dt}=s_{R2}^{B}-k_{int,R2}^{B} [R_{2}]_{B,i}-k_{on,V165,R2}^{B}[V_{165}]_{B}[R_{2}]_{B,i}+k_{off,V165R2}^{B}[V_{165}R_{2}]_{B,i} \\ -k_{on,V165b,R2}^{B}[V_{165b}]_{B}[R_{2}]_{B,i}+k_{off,V165bR2}^{B}[V_{165b}R_{2}]_{B,i} \\ -k_{on,V121,R2}^{B}[V_{121}]_{B}[R_{2}]_{B,i}+k_{off,V121R2}^{B}[V_{121}R_{2}]_{B,i} \\ -k_{c,V165N1,R2}^{B}[V_{165}N_{1}]_{B}[R_{2}]_{B,i}+k_{off,V165N1,R2}^{B}[R_{2}V_{165}N_{1}]_{B,i} \\ -k_{on,V165,R2}^{B}[\alpha2M\cdot V_{165}]_{B}[R_{2}]_{B,i}+k_{off,V165R2}^{B}[\alpha2M\cdot V_{165}R_{2}]_{B,i} \\ -k_{on,V165b,R2}^{B}[\alpha2M\cdot V_{165b}]_{B}[R_{2}]_{B,i}+k_{off,V165bR2}^{B}[\alpha2M\cdot V_{165b}R_{2}]_{B,i} \\ -k_{on,V121,R2}^{B}[\alpha2M\cdot V_{121}]_{B}[R_{2}]_{B,i}+k_{off,V121R2}^{B}[\alpha2M\cdot V_{121}R_{2}]_{B,i} \\ -k_{c,V165N1,R2}^{B}[\alpha2M\cdot V_{165}N_{1}]_{B}[R_{2}]_{B,i}+k_{off,V165N1,R2}^{B}[\alpha2M\cdot R_{2}V_{165}N_{1}]_{B,i} \\ -k_{on,V121,R2}^{B}[\alpha2M_{fast}\cdot V_{121}]_{B}[R_{2}]_{B,i}+k_{off,V121R2}^{B}[\alpha2M_{fast}\cdot V_{121}R_{2}]_{B,i} \\ -k_{on,V165,R2}^{B}[\alpha2M_{fast}\cdot V_{165}]_{B}[R_{2}]_{B,i}+k_{off,V165R2}^{B}[\alpha2M_{fast}\cdot V_{165}R_{2}]_{B,i} \\ -k_{on,V165b,R2}^{B}[\alpha2M_{fast}\cdot V_{165b}]_{B}[R_{2}]_{B,i}+k_{off,V165bR2}^{B}[\alpha2M_{fast}\cdot V_{165b}R_{2}]_{B,i} \\ -k_{c,V165N1,R2}^{B}[\alpha2M_{fast}\cdot V_{165}N_{1}]_{B}[R_{2}]_{B,i}+k_{off,V165N1,R2}^{B}[\alpha2M_{fast}\cdot R_{2}V_{165}N_{1}]_{B,i} \end{matrix}$ (S. 36)

$\begin{matrix} \frac{d[N_{1}]_{B,i}}{dt}=s_{N1}^{B}-k_{int,N1}^{B}[N_{1}]_{B,i}-k_{on,V165,N1}^{B}[V_{165}]_{B}[N_{1}]_{B,i}+k_{off,V165N1}^{B}[V_{165}N_{1}]_{B,i} \\ -k_{c,V121R1,N1}^{B}[V_{121}R_{1}]_{B}[N_{1}]_{B,i}+k_{dissoc,R1N1}^{B}[V_{121}R_{1}N_{1}]_{B,i} \\ -k_{c,V165R2,N1}^{B}[V_{165}R_{2}]_{B,i}[N_{1}]_{B,i}+k_{off,V165R2,N1}^{B}[R_{2}V_{165}N_{1}]_{B,i} \\ -k_{c,R1,N1}^{B}[N_{1}]_{B,i}[R_{1}]_{B,i}+k_{dissoc,R1N1}^{B}[R_{1}N_{1}]_{B,i} \\ -k_{c,V121R1,N1}^{B}[\alpha2M\cdot V_{121}R_{1}]_{B}[N_{1}]_{B,i}+k_{dissoc,\mathrm{V}121R1N1}^{B}[\alpha2M\cdot V_{121}R_{1}N_{1}]_{B,i} \\ -k_{on,V165,N1}^{B}[\alpha2M\cdot V_{165}]_{B}[N_{1}]_{B,i}+k_{off,V165N1}^{B}[\alpha2M\cdot V_{165}N_{1}]_{B,i} \\ -k_{c,V165R2,N1}^{B}[\alpha2M\cdot V_{165}R_{2}]_{B,i}[N_{1}]_{B,i}+k_{off,V165R2N1}^{B}[\alpha2M\cdot R_{2}V_{165}N_{1}]_{B,i} \\ -k_{c,V121R1,N1}^{B}[\alpha2M_{fast}\cdot V_{121}R_{1}]_{B}[N_{1}]_{B,i}+k_{dissoc,\mathrm{V}121R1N1}^{B}[\alpha2M_{fast}\cdot V_{121}R_{1}N_{1}]_{B,i} \\ -k_{on,V165,N1}^{B}[\alpha2M_{fast}\cdot V_{165}]_{B}[N_{1}]_{B,i}+k_{off,V165N1}^{B}[\alpha2M_{fast}\cdot V_{165}N_{1}]_{B,i} \\ -k_{c,V165R2,N1}^{B}[\alpha2M_{fast}\cdot V_{165}R_{2}]_{B,i}[N_{1}]_{B,i}+k_{off,V165R2N1}^{B}[\alpha2M_{fast}\cdot R_{2}V_{165}N_{1}]_{B,i} \end{matrix}$ (S. 37)

$\frac{d[V_{165}R_{1}]_{B,i}}{dt}=-k_{int,V165R1}^{B}[V_{165}R_{1}]_{B,i}+k_{on,V165,R1}^{B}[V_{165}]_{B}[R_{1}]_{B,i}-k_{off,V165R1}^{B}[V_{165}R_{1}]_{B,i}$ (S. 38)

$\frac{d[V_{165}R_{2}]_{B,i}}{dt}=-k_{int,V165R2}^{B}[V_{165}R_{2}]_{B,i}+k_{on,V165,R2}^{B}[V_{165}]_{B}[R_{2}]_{B,i}-k_{off,V165R2}^{B}[V_{165}R_{2}]_{B,i}$ (S. 39)

$\frac{d[V_{165b}R_{1}]_{B,i}}{dt}=-k_{int,V165bR1}^{B}[V_{165b}R_{1}]_{B,i}+k_{on,V165b,R1}^{B}[V_{165b}]_{B}[R_{1}]_{B,i}-k_{off,V165bR1}^{B}[V_{165b}R_{1}]_{B,i}$ (S. 40)

$\frac{d[V_{165b}R_{2}]_{B,i}}{dt}=-k_{int,V165bR2}^{B}[V_{165b}R_{2}]_{B,i}+k_{on,V165b,R2}^{B}[V_{165b}]_{B}[R_{2}]_{B,i}-k_{off,V165bR2}^{B}[V_{165b}R_{2}]_{B,i}$ (S. 41)

$\begin{matrix} \frac{d[V_{165}N_{1}]_{B,i}}{dt}=-k_{int,V165N1}^{B}[V_{165}N_{1}]_{B,i}+k_{on,V165,N1}^{B}[V_{165}]_{B}[N_{1}]_{B,i}-k_{off,V165N1}^{B}[V_{165}N_{1}]_{B,i} \\ -k_{c,V165N1,R2}^{B}[V_{165}N_{1}]_{B,i}[R_{2}]_{B,i}+k_{off,V165N1R2}^{B}[R_{2}V_{165}N_{1}]_{B,i} \end{matrix}$ (S. 42)

$\begin{matrix} \frac{d[R_{2}V_{165}N_{1}]_{B,i}}{dt}=-k_{int,V165R2N1}^{B}[R_{2}V_{165}N_{1}]_{B,i} \\ +k_{c,V165R2,N1}^{B}[V_{165}R_{2}]_{B,i}[N_{1}]_{B,i}-k_{off,V165R2N1}^{B}[R_{2}V_{165}N_{1}]_{B,i} \\ +k_{c,V165N1,R2}^{B}[V_{165}N_{1}]_{B,i}[R_{2}]_{B,i}-k_{off,V165N1R2}^{B}[R_{2}V_{165}N_{1}]_{B,i} \end{matrix}$ (S. 43)

$\begin{matrix} \frac{d[V_{121}R_{1}]_{B,i}}{dt}=-k_{int,V121R1}^{B}[V_{121}R_{1}]_{B,i} \\ +k_{on,V121,R1}^{B}[V_{121}]_{B,i}[R_{1}]_{B,i}-k_{off,V121R1}^{B}[V_{121}R_{1}]_{B,i} \\ -k_{c,R1,N1}^{B}[V_{121}R_{1}]_{B,i}[N_{1}]_{B,i}+k_{dissoc,R1N1}^{B}[V_{121}R_{1}N_{1}]_{B,i} \end{matrix}$ (S. 44)

$\frac{d[V_{121}R_{2}]_{B,i}}{dt}=-k_{int,V121R2}^{B}[V_{121}R_{2}]_{B,i}+k_{on,V121,R2}^{B}[V_{121}]_{B}[R_{2}]_{B,i}-k_{off,V121R2}^{B}[V_{121}R_{2}]_{B,i}$ (S. 45)

$\begin{matrix} \frac{d[V_{121}R_{1}N_{1}]_{B,i}}{dt}=-k_{intV121R1N1}^{B}[V_{121}R_{1}N_{1}]_{B,i}\text{ } \\ +k_{c,V121R1,N1}^{B}[V_{121}R_{1}]_{B,i}[N_{1}]_{B,i}-k_{dissoc,V121N1}^{B}[V_{121}R_{1}N_{1}]_{B,i} \\ +k_{on,V121R1N1}^{B}[V_{121}]_{B}[R_{1}N_{1}]_{B,i}-k_{off,V121R1N1}^{B}[V_{121}R_{1}N_{1}]_{B,i} \end{matrix}$ (S. 46)

$\begin{matrix} \frac{d[R_{1}N_{1}]_{B,i}}{dt}=-k_{int,R1N1}^{B}[R_{1}N_{1}]_{B,i} \\ +k_{c,R1,N1}^{B}[R_{1}]_{B,i}[N_{1}]_{B,i}-k_{dissoc,R1N1}^{B}[R_{1}N_{1}]_{B,i} \\ -k_{on,V121,R1}^{B}[V_{121}]_{B}[R_{1}N_{1}]_{B,i}+k_{off,V121R1}^{B}[V_{121}R_{1}N_{1}]_{B,i} \end{matrix}$ (S. 47)

$\begin{matrix} \frac{d[sR_{1}N_{1}]_{B,i}}{dt}=-k_{int,sR1N1}^{B}[sR_{1}N_{1}]_{B,i}+k_{on,sR1,N1}^{B}[sR_{1}]_{B}[N_{1}]_{B,i}-k_{off,sR1N1}^{B}[sR_{1}N_{1}]_{B,i} \\ -k_{on,V121,sR1N1}^{B}[V_{121}]_{B}[sR_{1}N_{1}]_{B,i}+k_{off,V121,sR1N1}^{B}[V_{121}sR_{1}N_{1}]_{B,i} \end{matrix}$ (S. 48)

$\begin{matrix} \frac{d[V_{121}sR_{1}N_{1}]_{B,i}}{dt}=-k_{int,V121sR1N1}^{B}[V_{121}sR_{1}N_{1}]_{B,i} \\ +k_{on,sR1,N1}^{B}[V_{121}R_{1}]_{B}[N_{1}]_{B,i}-k_{off,sR1N1}^{B}[V_{121}sR_{1}N_{1}]_{B,i} \\ +k_{on,V121sR1N1}^{B}[V_{121}]_{B}[R_{1}N_{1}]_{B,i}-k_{off,V121sR1N1}^{B}[V_{121}sR_{1}N_{1}]_{B,i} \end{matrix}$ (S. 49)

$\frac{d[\alpha2M\cdot V_{165}R_{1}]_{B,i}}{dt}=k_{on,V165,R1}^{B}[\alpha2M\cdot V_{165}]_{B}[R_{1}]_{B,i}-k_{off,V165R1}^{B}[\alpha2M\cdot V_{165}R_{1}]_{B,i}$ (S. 50)

$\frac{d[\alpha2M\cdot V_{165}R_{2}]_{B,i}}{dt}=k_{on,V165,R2}^{B}[\alpha2M\cdot V_{165}]_{B}[R_{2}]_{B,i}-k_{off,V165R2}^{B}[\alpha2M\cdot V_{165}R_{2}]_{B,i}$ (S. 51)

$\frac{d[\alpha2M\cdot V_{165b}R_{1}]_{B,i}}{dt}=k_{on,V165b,R1}^{B}[\alpha2M\cdot V_{165b}]_{B}[R_{1}]_{B,i}-k_{off,V165bR1}^{B}[\alpha2M\cdot V_{165b}R_{1}]_{B,i}$ (S. 52)

$\frac{d[\alpha2M\cdot V_{165b}R_{2}]_{B,i}}{dt}=k_{on,V165b,R2}^{B}[\alpha2M\cdot V_{165b}]_{B}[R_{2}]_{B,i}-k_{off,V165bR2}^{B}[\alpha2M\cdot V_{165b}R_{2}]_{B,i}$ (S. 53)

$\begin{matrix} \frac{d[\alpha2M\cdot V_{165}N_{1}]_{B,i}}{dt}=k_{on,V165,N1}^{B}[\alpha2M\cdot V_{165}]_{B}[N_{1}]_{B,i}-k_{off,V165N1}^{B}[\alpha2M\cdot V_{165}N_{1}]_{B,i} \\ -k_{c,V165N1,R2}^{B}[\alpha2M\cdot V_{165}N_{1}]_{B,i}[R_{2}]_{B,i}+k_{off,V165N1R2}^{B}[\alpha2M\cdot R_{2}V_{165}N_{1}]_{B,i} \end{matrix}$ (S. 54)

$\begin{matrix} \frac{d[\alpha2M\cdot R_{2}V_{165}N_{1}]_{B,i}}{dt}=k_{c,V165R2,N1}^{B}[\alpha2M\cdot V_{165}R_{2}]_{B,i}[N_{1}]_{B,i}-k_{off,V165R2N1}^{B}[\alpha2M\cdot R_{2}V_{165}N_{1}]_{B,i} \\ +k_{c,V165N1,R2}^{B}[\alpha2M\cdot V_{165}N_{1}]_{B,i}[R_{2}]_{B,i}-k_{off,V165N1R2}^{B}[\alpha2M\cdot R_{2}V_{165}N_{1}]_{B,i} \end{matrix}$ (S. 55)

$\begin{matrix} \frac{d[\alpha2M\cdot V_{121}R_{1}]_{B,i}}{dt}=k_{on,V121,R1}^{B}[\alpha2M\cdot V_{121}]_{B,i}[R_{1}]_{B,i}-k_{off,V121R1}^{B}[\alpha2M\cdot V_{121}R_{1}]_{B,i} \\ -k_{c,R1,N1}^{B}[\alpha2M\cdot V_{121}R_{1}]_{B,i}[N_{1}]_{B,i}+k_{dissoc,R1N1}^{B}[\alpha2M\cdot V_{121}R_{1}N_{1}]_{B,i} \end{matrix}$ (S. 56)

$\frac{d[\alpha2M\cdot V_{121}R_{2}]_{B,i}}{dt}=k_{on,V121,R2}^{B}[\alpha2M\cdot V_{121}]_{B}[R_{2}]_{B,i}-k_{off,V121R2}^{B}[\alpha2M\cdot V_{121}R_{2}]_{B,i}$ (S. 57)

$\begin{matrix} \frac{d[\alpha2M\cdot V_{121}R_{1}N_{1}]_{B,i}}{dt}=k_{c,V121R1,N1}^{B}[\alpha2M\cdot V_{121}R_{1}]_{B,i}[N_{1}]_{B,i}-k_{dissoc,V12N1}^{B}[\alpha2M\cdot V_{121}R_{1}N_{1}]_{B,i} \\ +k_{on,V121R1N1}^{B}[\alpha2M\cdot V_{121}]_{B}[R_{1}N_{1}]_{B,i}-k_{off,V121R1N1}^{B}[\alpha2M\cdot V_{121}R_{1}N_{1}]_{B,i} \end{matrix}$ (S. 58)

$\frac{d[\alpha2M_{fast}\cdot V_{165}R_{1}]_{B,i}}{dt}=k_{on,V165,R1}^{B}[\alpha2M_{fast}\cdot V_{165}]_{B}[R_{1}]_{B,i}-k_{off,V165R1}^{B}[\alpha2M_{fast}\cdot V_{165}R_{1}]_{B,i}$ (S. 59)

$\frac{d[\alpha2M_{fast}\cdot V_{165b}R_{1}]_{B,i}}{dt}=k_{on,V165b,R1}^{B}[\alpha2M_{fast}\cdot V_{165b}]_{B}[R_{1}]_{B,i}-k_{off,V165bR1}^{B}[\alpha2M_{fast}\cdot V_{165b}R_{1}]_{B,i}$ (S. 60)

$\begin{matrix} \frac{d[\alpha2M_{fast}\cdot V_{165}N_{1}]_{B,i}}{dt}=k_{on,V165,N1}^{B}[\alpha2M_{fast}\cdot V_{165}]_{B}[N_{1}]_{B,i}-k_{off,V165N1}^{B}[\alpha2M_{fast}\cdot V_{165}N_{1}]_{B,i} \\ -k_{c,V165N1,R2}^{B}[\alpha2M_{fast}\cdot V_{165}N_{1}]_{B,i}[R_{2}]_{B,i}+k_{off,V165N1R2}^{B}[\alpha2M_{fast}\cdot R_{2}V_{165}N_{1}]_{B,i} \end{matrix}$ (S. 61)

$\begin{matrix} \frac{d[\alpha2M_{fast}\cdot R_{2}V_{165}N_{1}]_{B,i}}{dt}=k_{c,V165R2,N1}^{B}[\alpha2M_{fast}\cdot V_{165}R_{2}]_{B,i}[N_{1}]_{B,i}-k_{off,V165R2N1}^{B}[\alpha2M_{fast}\cdot R_{2}V_{165}N_{1}]_{B,i} \\ +k_{c,V165N1,R2}^{B}[\alpha2M_{fast}\cdot V_{165}N_{1}]_{B,i}[R_{2}]_{B,i}-k_{off,V165N1R2}^{B}[\alpha2M_{fast}\cdot R_{2}V_{165}N_{1}]_{B,i} \end{matrix}$ (S. 62)

$\begin{matrix} \frac{d[\alpha2M_{fast}\cdot V_{121}R_{1}]_{B,i}}{dt}=k_{on,V121,R1}^{B}[\alpha2M_{fast}\cdot V_{121}]_{B,i}[R_{1}]_{B,i}-k_{off,V121R1}^{B}[\alpha2M_{fast}\cdot V_{121}R_{1}]_{B,i} \\ -k_{c,R1,N1}^{B}[\alpha2M_{fast}\cdot V_{121}R_{1}]_{B,i}[N_{1}]_{B,i}+k_{dissoc,R1N1}^{B}[\alpha2M_{fast}\cdot V_{121}R_{1}N_{1}]_{B,i} \end{matrix}$ (S. 63)

$\frac{d[\alpha2M_{fast}\cdot V_{121}R_{2}]_{B,i}}{dt}=k_{on,V121,R2}^{B}[\alpha2M_{fast}\cdot V_{121}]_{B}[R_{2}]_{B,i}-k_{off,V121R2}^{B}[\alpha2M_{fast}\cdot V_{121}R_{2}]_{B,i}$ (S. 64)

$$\begin{matrix} \frac{d[\alpha2M_{fast}\cdot V_{121}R_{1}N_{1}]_{B,i}}{dt}=k_{c,V121R1,N1}^{B}[\alpha2M_{fast}\cdot V_{121}R_{1}]_{B,i}[N_{1}]_{B,i}-k_{dissoc,V121N1}^{B}[\alpha2M_{fast}\cdot V_{121}R_{1}N_{1}]_{B,i} \\ +k_{on,V121R1N1}^{B}[\alpha2M_{fast}\cdot V_{121}]_{B}[R_{1}N_{1}]_{B,i}-k_{off,V121R1N1}^{B}[\alpha2M_{fast}\cdot V_{121}R_{1}N_{1}]_{B,i} \end{matrix}$$

(S. 65)

II. Plasma

$\begin{matrix} \frac{d[V_{165}]_{B}}{dt}=q_{V165}^{B}-c_{V165}[V_{165}]_{B}-k_{on,V165,R1}^{B}[V_{165}]_{B}[R_{1}]_{B,i}+k_{off,V165R1}^{B}[V_{165}R_{1}]_{B,i} \\ -k_{on,V165,R2}^{B}[V_{165}]_{B}[R_{2}]_{B,i}+k_{off,V165R2}^{B}[V_{165}R_{2}]_{B,i} \\ -k_{on,V165,N1}^{B}[V_{165}]_{B}[N_{1}]_{B,i}+k_{off,V165N1}^{B}[V_{165}N_{1}]_{B,i} \\ -k_{on,V165,\alpha2M}^{B}[V_{165}]_{B}[\alpha2M]_{B}+k_{off,V165\alpha2M}^{B}[\alpha2M\cdot V_{165}]_{B} \\ -k_{on,V165,\alpha2Mfast}^{B}[V_{165}]_{B}[\alpha2M_{fast}]_{B}+k_{off,V165\alpha2Mfast}^{B}[\alpha2M_{fast}\cdot V_{165}]_{B} \\ -\frac{k_{p,V}^{BN}S_{NB}}{U_{p}}[V_{165}]_{B}+\left(\frac{k_{L}+k_{p,V}^{NB}S_{NB}}{U_{B}}\right)\frac{[V_{165}]_{N}}{K_{AV,N}} \\ -\frac{k_{p,V}^{BD}S_{DB}}{U_{p}}[V_{165}]_{B}+\left(\frac{k_{L}+k_{p,V}^{DB}S_{DB}}{U_{B}}\right)\frac{[V_{165}]_{D}}{K_{AV,\mathrm{D}}} \end{matrix}$ (S. 66)

$\begin{matrix} \frac{d[V_{165b}]_{B}}{dt}=q_{V165b}^{B}-c_{V165b}[V_{165b}]_{B} \\ -k_{on,V165b,R1}^{B}[V_{165b}]_{B}[R_{1}]_{B,i}+k_{off,V165bR1}^{B}[V_{165b}R_{1}]_{B,i} \\ -k_{on,V165b,R2}^{B}[V_{165b}]_{B}[R_{2}]_{B,i}+k_{off,V165bR2}^{B}[V_{165b}R_{2}]_{B,i} \\ -k_{on,V165b,\alpha2M}^{B}[V_{165b}]_{B}[\alpha2M]_{B}+k_{off,V165b\alpha2M}^{B}[\alpha2M\cdot V_{165b}]_{B} \\ -k_{on,V165b,\alpha2Mfast}^{B}[V_{165b}]_{B}[\alpha2M_{fast}]_{B}+k_{off,V165b\alpha2Mfast}^{B}[\alpha2M_{fast}\cdot V_{165b}]_{B} \\ -\frac{k_{p,V}^{BN}S_{NB}}{U_{p}}[V_{165b}]_{B}+\left(\frac{k_{L}+k_{p,V}^{NB}S_{NB}}{U_{B}}\right)\frac{[V_{165b}]_{N}}{K_{AV,N}} \\ -\frac{k_{p,V}^{BD}S_{DB}}{U_{p}}[V_{165b}]_{B}+\left(\frac{k_{L}+k_{p,V}^{DB}S_{DB}}{U_{B}}\right)\frac{[V_{165b}]_{D}}{K_{AV,\mathrm{D}}} \end{matrix}$ (S. 67)

$\begin{matrix} \frac{d[V_{121}]_{B}}{dt}=q_{V121}^{B}-c_{V121}[V_{121}]_{B}-k_{on,V121,R1}^{B}[V_{121}]_{B}[R_{1}]_{B,i}+k_{off,V121R1}^{B}[V_{121}R_{1}]_{B,i} \\ -k_{on,V121,R1N1}^{B}[V_{121}]_{B}[R_{1}N_{1}]_{B,i}+k_{off,V121R1N1}^{B}[V_{121}R_{1}N_{1}]_{B,i} \\ -k_{on,V121,R2}^{B}[V_{121}]_{B}[R_{2}]_{B,i}+k_{off,V121R2}^{B}[V_{121}R_{2}]_{B,i} \\ -k_{on,V121,\alpha2M}^{B}[V_{121}]_{B}[\alpha2M]_{B}+k_{off,V121\alpha2M}^{B}[\alpha2M\cdot V_{121}]_{B} \\ -k_{on,V121,\alpha2Mfast}^{B}[V_{121}]_{B}[\alpha2M_{fast}]_{B}+k_{off,V121\alpha2Mfast}^{B}[\alpha2M\cdot V_{121}]_{B} \\ -\frac{k_{p,V}^{BN}S_{NB}}{U_{p}}[V_{121}]_{B}+\left(\frac{k_{L}+k_{p,V}^{NB}S_{NB}}{U_{B}}\right)\frac{[V_{121}]_{N}}{K_{AV,N}} \\ -\frac{k_{p,V}^{BD}S_{DB}}{U_{p}}[V_{121}]_{B}+\left(\frac{k_{L}+k_{p,V}^{DB}S_{DB}}{U_{B}}\right)\frac{[V_{121}]_{D}}{K_{AV,\mathrm{D}}} \end{matrix}$ (S. 68)

$\begin{matrix} \frac{d[sR_{1}]_{B}}{dt}=q_{sR1}^{B}-\left(c_{sR1}+k_{\deg,sR1}\right)[sR_{1}]_{B} \\ -k_{on,V165,sR1}^{B}[V_{165}]_{B}[sR_{1}]_{B}+k_{off,V165sR1}^{B}[V_{165}sR_{1}]_{B} \\ -k_{on,V165b,sR1}^{B}[V_{165b}]_{B}[sR_{1}]_{B}+k_{off,V165bsR1}^{B}[V_{165b}sR_{1}]_{B} \\ -k_{on,V121,sR1}^{B}[V_{121}]_{B}[sR_{1}]_{B}+k_{off,V121sR1}^{B}[V_{121}sR_{1}]_{B} \\ -k_{on,sR1,N1}^{B}[sR_{1}]_{B}[N_{1}]_{B,i}+k_{off,sR1N1}^{B}[sR_{1}N_{1}]_{B,i} \\ -k_{p,sR1}^{BN}\frac{S_{NB}}{U_{p}}[sR_{1}]_{B}+\left(\frac{k_{L}+k_{p,sR1}^{NB}S_{NB}}{U_{B}}\right)\frac{[sR_{1}]_{N}}{K_{AV,N}} \\ -k_{p,sR1}^{BD}\frac{S_{DB}}{U_{p}}[sR_{1}]_{B}+\left(\frac{k_{L}+k_{p}^{DB}S_{DB}}{U_{B}}\right)\frac{[sR_{1}]_{D}}{K_{AV,\mathrm{D}}} \end{matrix}$ (S. 69)

$\begin{matrix} \frac{d[V_{165}sR_{1}]_{B}}{dt}=-\left(c_{sR1}+k_{\deg,VsR1}\right)[V_{165}sR_{1}]_{B} \\ +k_{on,V165,sR1}^{B}[V_{165}]_{B}[sR_{1}]_{B}-k_{off,V165sR1}^{B}[V_{165}sR_{1}]_{B} \\ -k_{p,sR1}^{BN}\frac{S_{NB}}{U_{p}}[V_{165}sR_{1}]_{B}+\left(\frac{k_{L}+k_{p,sR1}^{NB}S_{NB}}{U_{B}}\right)\frac{[V_{165}sR_{1}]_{N}}{K_{AV,N}} \\ -k_{p,sR1}^{BD}\frac{S_{DB}}{U_{p}}[V_{165}sR_{1}]_{B}+\left(\frac{k_{L}+k_{p}^{DB}S_{DB}}{U_{B}}\right)\frac{[V_{165}sR_{1}]_{D}}{K_{AV,\mathrm{D}}} \end{matrix}$ (S. 70)

$\begin{matrix} \frac{d[V_{165b}sR_{1}]_{B}}{dt}=-\left(c_{sR1}+k_{\deg,VsR1}\right)[V_{165b}sR_{1}]_{B} \\ +k_{on,V165b,sR1}^{B}[V_{165b}]_{B}[sR_{1}]_{B}-k_{off,V165bsR1}^{B}[V_{165b}sR_{1}]_{B} \\ -k_{p,sR1}^{BN}\frac{S_{NB}}{U_{p}}[V_{165b}sR_{1}]_{B}+\left(\frac{k_{L}+k_{p,sR1}^{NB}S_{NB}}{U_{B}}\right)\frac{[V_{165b}sR_{1}]_{N}}{K_{AV,N}} \\ -k_{p,sR1}^{BD}\frac{S_{DB}}{U_{p}}[V_{165b}sR_{1}]_{B}+\left(\frac{k_{L}+k_{p}^{DB}S_{DB}}{U_{B}}\right)\frac{[V_{165b}sR_{1}]_{D}}{K_{AV,\mathrm{D}}} \end{matrix}$ (S. 71)

$\begin{matrix} \frac{d[V_{121}sR_{1}]_{B}}{dt}=-\left(c_{sR1}+k_{\deg,VsR1}\right)[V_{121}sR_{1}]_{B} \\ +k_{on,V121,sR1}^{B}[V_{121}]_{B}[sR_{1}]_{B}-k_{off,V121sR1}^{B}[V_{121}sR_{1}]_{B} \\ -k_{on,sR1,N1}^{B}[V_{121}sR_{1}]_{B}[N_{1}]_{B,i}+k_{off,R1sN1}^{B}[V_{121}sR_{1}N_{1}]_{B,i} \\ -k_{p,sR1}^{BN}\frac{S_{NB}}{U_{p}}[V_{121}sR_{1}]_{B}+\left(\frac{k_{L}+k_{p,sR1}^{NB}S_{NB}}{U_{B}}\right)\frac{[V_{121}sR_{1}]_{N}}{K_{AV,N}} \\ -k_{p,sR1}^{BD}\frac{S_{DB}}{U_{p}}[V_{121}sR_{1}]_{B}+\left(\frac{k_{L}+k_{p}^{DB}S_{DB}}{U_{B}}\right)\frac{[V_{121}sR_{1}]_{D}}{K_{AV,\mathrm{D}}} \end{matrix}$ (S. 72)

$\begin{matrix} \frac{d[\alpha2M]_{B}}{dt}=k_{syn,\alpha2M}-c_{\alpha2M}[\alpha2M]_{B} \\ -k_{on,\alpha2M,V165}^{B}[\alpha2M]_{B}[V_{165}]_{B}+k_{off,\alpha2MV165}^{B}[\alpha2M\cdot V_{165}]_{B} \\ -k_{on,\alpha2M,V165b}^{B}[\alpha2M]_{B}[V_{165b}]_{B}+k_{off,\alpha2MV165b}^{B}[\alpha2M\cdot V_{165b}]_{B} \\ -k_{on,\alpha2M,V121}^{B}[\alpha2M]_{B}[V_{121}]_{B}+k_{off,\alpha2MV121}^{B}[\alpha2M\cdot V_{121}]_{B} \end{matrix}$ (S. 73)

$\begin{matrix} \frac{d[\alpha2M_{fast}]_{B}}{dt}=k_{syn,\alpha2Mfast}-c_{\alpha2Mfast}[\alpha2M_{fast}]_{B} \\ -k_{on,\alpha2M,V165}^{B}[\alpha2M_{fast}]_{B}[V_{165}]_{B}+k_{off,\alpha2MV165}^{B}[\alpha2M_{fast}\cdot V_{165}]_{B} \\ -k_{on,\alpha2M,V165b}^{B}[\alpha2M_{fast}]_{B}[V_{165b}]_{B}+k_{off,\alpha2MV165b}^{B}[\alpha2M_{fast}\cdot V_{165b}]_{B} \\ -k_{on,\alpha2M,V121}^{B}[\alpha2M_{fast}]_{B}[V_{121}]_{B}+k_{off,\alpha2MV121}^{B}[\alpha2M_{fast}\cdot V_{121}]_{B} \end{matrix}$ (S. 74)

$\begin{matrix} \frac{d[\alpha2M\cdot V_{165}]_{B}}{dt}=-c_{\alpha2MV165}[\alpha2M\cdot V_{165}]_{B} \\ -k_{on,V165,R1}^{B}[\alpha2M\cdot V_{165}]_{B}[R_{1}]_{B,i}+k_{off,V165R1}^{B}[\alpha2M\cdot V_{165}R_{1}]_{B,i} \\ -k_{on,V165,R2}^{B}[\alpha2M\cdot V_{165}]_{B}[R_{2}]_{B,i}+k_{off,V165R2}^{B}[\alpha2M\cdot V_{165}R_{2}]_{B,i} \\ -k_{on,V165,N1}^{B}[\alpha2M\cdot V_{165}]_{B}[N_{1}]_{B,i}+k_{off,V165N1}^{B}[\alpha2M\cdot V_{165}N_{1}]_{B,i} \end{matrix}$ (S. 75)

$\begin{matrix} \frac{d[\alpha2M\cdot V_{165b}]_{B}}{dt}=-c_{\alpha2MV165b}[\alpha2M\cdot V_{165b}]_{B} \\ -k_{on,V165b,R1}^{B}[\alpha2M\cdot V_{165b}]_{B}[R_{1}]_{B,i}+k_{off,V165bR1}^{B}[\alpha2M\cdot V_{165b}R_{1}]_{B,i} \\ -k_{on,V165b,R2}^{B}[\alpha2M\cdot V_{165b}]_{B}[R_{2}]_{B,i}+k_{off,V165bR2}^{B}[\alpha2M\cdot V_{165b}R_{2}]_{B,i} \end{matrix}$ (S. 76)

$\begin{matrix} \frac{d[\alpha2M\cdot V_{121}]_{B}}{dt}=-c_{\alpha2MV121}[\alpha2M\cdot V_{121}]_{B} \\ -k_{on,V121,R1}^{B}[\alpha2M\cdot V_{121}]_{B}[R_{1}]_{B,i}+k_{off,V121R1}^{B}[\alpha2M\cdot V_{121}R_{1}]_{B,i} \\ -k_{on,V121,R1N1}^{B}[\alpha2M\cdot V_{121}]_{B}[R_{1}N_{1}]_{B,i}+k_{off,V121R1N1}^{B}[\alpha2M\cdot V_{121}R_{1}N_{1}]_{B,i} \\ -k_{on,V121,R2}^{B}[\alpha2M\cdot V_{121}]_{B}[R_{2}]_{B,i}+k_{off,V121R2}^{B}[\alpha2M\cdot V_{121}R_{2}]_{B,i} \end{matrix}$ (S. 77)

$\begin{matrix} \frac{d[\alpha2M_{fast}\cdot V_{165}]_{B}}{dt}=-c_{\alpha2MfastV165}[\alpha2M_{fast}\cdot V_{165}]_{B} \\ -k_{on,V165,R1}^{B}[\alpha2M_{fast}\cdot V_{165}]_{B}[R_{1}]_{B,i}+k_{off,V165R1}^{B}[\alpha2M_{fast}\cdot V_{165}R_{1}]_{B,i} \\ -k_{on,V165,R2}^{B}[\alpha2M_{fast}\cdot V_{165}]_{B}[R_{2}]_{B,i}+k_{off,V165R2}^{B}[\alpha2M_{fast}\cdot V_{165}R_{2}]_{B,i} \\ -k_{on,V165,N1}^{B}[\alpha2M_{fast}\cdot V_{165}]_{B}[N_{1}]_{B,i}+k_{off,V165N1}^{B}[\alpha2M_{fast}\cdot V_{165}N_{1}]_{B,i} \end{matrix}$ (S. 78)

$\begin{matrix} \frac{d[\alpha2M_{fast}\cdot V_{165b}]_{B}}{dt}=-c_{\alpha2MfastV165b}[\alpha2M_{fast}\cdot V_{165b}]_{B} \\ -k_{on,V165b,R1}^{B}[\alpha2M_{fast}\cdot V_{165b}]_{B}[R_{1}]_{B,i}+k_{off,V165bR1}^{B}[\alpha2M_{fast}\cdot V_{165b}R_{1}]_{B,i} \\ -k_{on,V165b,R2}^{B}[\alpha2M_{fast}\cdot V_{165b}]_{B}[R_{2}]_{B,i}+k_{off,V165bR2}^{B}[\alpha2M_{fast}\cdot V_{165b}R_{2}]_{B,i} \end{matrix}$ (S. 79)

$\begin{matrix} \frac{d[\alpha2M_{fast}\cdot V_{121}]_{B}}{dt}=-c_{\alpha2MfastV121}[\alpha2M_{fast}\cdot V_{121}]_{B} \\ -k_{on,V121,R1}^{B}[\alpha2M_{fast}\cdot V_{121}]_{B}[R_{1}]_{B,i}+k_{off,V121R1}^{B}[\alpha2M_{fast}\cdot V_{121}R_{1}]_{B,i} \\ -k_{on,V121,R1N1}^{B}[\alpha2M_{fast}\cdot V_{121}]_{B}[R_{1}N_{1}]_{B,i}+k_{off,V121R1N1}^{B}[\alpha2M_{fast}\cdot V_{121}R_{1}N_{1}]_{B,i} \\ -k_{on,V121,R2}^{B}[\alpha2M_{fast}\cdot V_{121}]_{B}[R_{2}]_{B,i}+k_{off,V121R2}^{B}[\alpha2M_{fast}\cdot V_{121}R_{2}]_{B,i} \\ -k_{on,V121,A}^{B}[\alpha2M_{fast}\cdot V_{121}]_{B}[A]_{B}+k_{off,V121A}^{B}[\alpha2M_{fast}\cdot V_{121}A]_{B} \end{matrix}$ (S. 80)

1. **Glossary**
2. **Concentrations**

*[V_165_], [V_165b_], [V_121_]* Concentration of unbound VEGF_165_, VEGF_165b_ and VEGF_121_

*[M_ECM_], [M_EBM_], [M_PBM_]* Concentration of VEGF binding sites in the ECM, EBM and PBM

*[V_i_M_ECM_], [V_i_M_EBM_], [V_i_M_PBM_]* Concentration of VEGF isoform *i* bound to the ECM, EBM and PBM, *i*=165, 165b

*[R­_1_], [R_2_]* Concentration of unoccupied VEGFR1 and VEGFR2

*[N_1_]* Concentration of unoccupied NRP1

*[R_1_N_1_]* Concentration of the VEGFR1-NRP1 complex

*[V_i_R_j_]* Concentration of VEGF isoform *i* bound to VEGFR *j*

*[V_i_N_1_]* Concentration of VEGF isoform *i* bound to NRP1, i=165 and 121

*[R_2_V_165_N_1_]* Concentration of the VEGFR2-VEGF_165_-NRP1 ternary complex

*[V_121_R_1_N_1_]* Concentration of the VEGF_121_-VEGFR1-NRP1 ternary complex

*[sR_1_]* Concentration of sVEGFR1

*[sR_1_M_ECM_], [sR_1_M_EBM_], [sR_1_M_PBM_]* Concentration of sVEGFR1bound to the ECM, EBM, and PBM

*[sR_1_N_1_]* Concentration of sVEGFR1bound to NRP1

*[V_i_ sR_1_]* Concentration of the VEGF isoform *i* bound to sVEGFR1, *i*=165, 165b, 121

*[V_121_sR_1_N_1_]* Concentration of the VEGF_121_-sVEGFR1-NRP1 ternary complex

*[α2M]* Concentration of alpha-2-macroglobulin (α2M)

*[α2M*·*V_i_]* Concentration of α2M bound to VEGF isoform *i, i*=165, 165b, 121

*[α2M*·*V_i_R_1_]* Concentration of α2M bound to VEGF isoform *i*-VEGFR1, *i*=165, 165b, 121

*[α2M*·*V_i_R_2_]* Concentration of α2M bound to VEGF isoform *i*-VEGFR2, *i*=165, 165b, 121

*[α2M*·*R_2_V_165_N_1_]* Concentration of α2M bound to the VEGFR2-VEGF_165_-NRP1 ternary complex

*[α2M*·*V_121_R_1_N_1_]* Concentration of α2M bound to the VEGF_121_-VEGFR1-NRP1 ternary complex

*[α2M_fast_]* Concentration of activated alpha-2-macroglobulin (α2M_fast_)

*[α2M_fast_* ·*V_i_]* Concentration of α2M_fast_ bound to VEGF isoform *i*, *i*=165, 165b, 121

*[α2M_fast_* ·*V_i_R_1_]* Concentration of α2M_fast_ bound to VEGF isoform *i*-VEGFR1, *i*=165, 165b, 121

*[α2M_fast_* ·*V_i_R_2_]* Concentration of α2M_fast_ bound to VEGF isoform *i*-VEGFR2, *i*=165, 165b, 121

*[α2M_fast_* ·*V_i_N_1_]* Concentration of α2M_fast_ bound to VEGF isoform *i*-NRP1, *i*=165, 121

*[α2M_fast_* ·*V_121_R_1_N_1_]* Concentration of α2M_fast_ bound to the VEGF isoform *i*-VEGFR1-NRP1 ternary complex, *i*=165, 121

*[α2M_fast_* ·*V_i_R_2_N_1_]* Concentration of α2M_fast_ bound to the VEGF isoform *i*-VEGFR2-NRP1 ternary complex, *i*=165, 121

1. **Geometric parameters**

*U_i_* Volume of compartment *i* (*N*=normal tissue, *B*=blood, *P*=plasma, *D*=calf muscle)

*S_iB_* Total surface area of endothelial cells at the interface of compartment *i* and blood (*N*=normal tissue, *T*=tumor)

*K_AV,i_* Available volume fraction in the tissue, i.e., ratio of available fluid volume to total tissue volume *U_i_*

1. **Kinetic parameters**

*q_V165_, q_V165b_, q_V121_* Secretion rate of VEGF_165_, VEGF_165b_ and VEGF_121_

*q_sR1_* Secretion rate of sVEGFR1

*k_on_* Kinetic binding rate

*k_off_* Kinetic unbinding rate

*k_c_* Kinetic coupling rate for receptors

*k_int_* Internalization rate of receptors

$k_{p,V}^{ij}$ Microvascular permeability of VEGF from compartment *i* to compartment *j* (*N*=normal tissue, *B*=blood, *D*=PAD calf)

*k_L_* Lymphatic drainage rate

*c_V165_, c_V165b_, c_V121_* Rate of plasma clearance of VEGF_165_, VEGF_165b_ and VEGF_121_

*k_deg,V_* Rate of degradation of VEGF isoforms

$k_{p,sR1}^{ij}$ Microvascular permeability of sVEGFR1 from compartment *i* to compartment *j* (*N*=normal tissue, *B*=blood, *D*=PAD calf)

$k_{p,VsR1}^{ij}$ Microvascular permeability of VEGF-sVEGFR1 complex from compartment *i* to compartment *j* (*N*=normal tissue, *B*=blood, *D=*PAD calf)

*k_deg,sR1_* Rate of degradation of sVEGFR1

*k_deg,VsR1_* Rate of degradation of VEGF-sVEGFR1 complex

*k _syn,α2M_* Rate of synthesis of α2M

*k _syn,α2Mfast_* Rate of synthesis of α2M_fast_

*c_α2M_* Rate of plasma clearance of α2M

*c_α2MV_* Rate of plasma clearance of α2M-VEGF complex

*c_α2Mfast_* Rate of plasma clearance of α2M_fast_

*c_α2MfastV_* Rate of plasma clearance of α2M_fast_-VEGF complex
